# Antidepressant-like Properties of a Selenium-Containing Pyridinium Salt Explored through In Vitro, In Vivo, and In Silico Approaches

**DOI:** 10.1021/acschemneuro.5c00233

**Published:** 2025-09-12

**Authors:** Mariana Parron Paim, Taís da Silva Teixeira Rech, Letícia Devantier Krüger, Larissa Sander Magalhães, Filipe Penteado, Caroline Signorini Gomes, Eder João Lenardão, César Augusto Brüning, Cristiani Folharini Bortolatto

**Affiliations:** † Postgraduate Program in Biochemistry and Bioprospecting, Laboratory of Biochemistry and Molecular Neuropharmacology (LABIONEM), Center for Chemical, Pharmaceutical and Food Sciences (CCQFA), Federal University of Pelotas (UFPel), 96010-900 Pelotas, Rio Grande do Sul, Brazil; ‡ Center for Natural and Exact Sciences−CCNE, Federal University of Santa Maria−UFSM, Roraima Avenue, Building 18, 97105-340 Santa Maria, Rio Grande do Sul, Brazil; § Postgraduate Program in Chemistry (PPGQ), Clean Organic Synthesis Laboratory (LASOL), Center for Chemical, Pharmaceutical and Food Sciences (CCQFA), Federal University of Pelotas (UFPel), P.O. Box 354, 96010-900 Pelotas, Rio Grande do Sul, Brazil

**Keywords:** pyridinium salts, selenium, antioxidant, monoamine oxidase inhibitor, antidepressant, toxicity, mice

## Abstract

Selenium (Se) compounds have demonstrated antioxidant and antidepressant-like effects; however, most reported molecules are highly lipophilic. In contrast, moderate water solubility is considered crucial for drug delivery and therapeutic application. Accordingly, Se-containing pyridinium salts emerge as promising candidates for depression treatment. In this study, we conducted a comprehensive evaluation of three Se-based pyridinium salts (designated as compounds **3A**, **3B**, and **3C**) using in vitro, in vivo, and in silico approaches. All three compounds exhibited cerebral antioxidant activity, significantly reducing lipid peroxidation and protein carbonylation in vitro. They also demonstrated in vitro inhibition of monoamine oxidase A and B in mouse brain tissue. Subsequently, an in vivo investigation with the salts using the tail suspension test in male Swiss mice (single intragastric dose of 5 mg/kg) identified compound **3B** as the most effective antidepressant-like agent. Further dose-response (0.5–5 mg/kg) and time-response (15–120 min) analyses established that the minimum effective dose was 1 mg/kg administered over 30 min. In silico ADMET predictions indicated favorable pharmacokinetic properties, and an acute oral toxicity study revealed that a 50-fold higher dose (50 mg/kg) than the therapeutic level did not produce any observable adverse effects. Taken together, these findings suggest that compound **3B** represents a promising antidepressant candidate for future studies.

## Graphical abstract

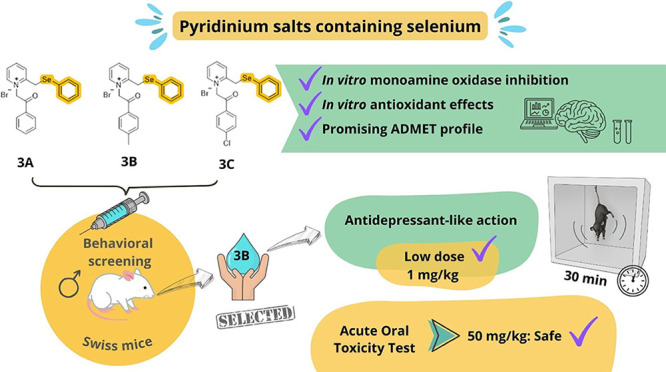

## 1 Introduction

Depressive disorder is a prevalent and enduring mental illness characterized by recurring symptoms of sadness, anhedonia, cognitive impairments, lack of energy, sleep disturbances, and changes in appetite.^1^ According to the World Health Organization (WHO), depression has emerged as the leading cause of disability globally.^2^ The worldwide prevalence rate of self-reported depressive symptoms has reached 34%.^3^ Until 2019, depression ranked 11th in the “Disability Adjusted Life Years” (DALY) index and third in terms of years of life lost due to disability in the Americas.^4^

Numerous drugs are available on the market to treat this disorder. However, the delayed onset of action and adverse effects often lead many patients to discontinue treatment.^5^ The likelihood of recurrence is high, after the initial episode of depression, approximately half of the patients experience a relapse or recurrence of the depressive symptoms.^6^ With each subsequent episode, the risk of recurrence increases, with nearly 80% of patients experiencing at least one episode in their lifetime.^7^ Additionally, 50% of patients with major depressive disorder exhibit resistance to treatment.^8^ These findings underscore the need for exploring new therapeutic approaches.

The need for new drugs and the trend in molecular hybridization have resulted in the development of many drug candidates, but most of them have greater lipophilicity, high molecular weight and low water solubility. Indeed, several commercialized medicines have low solubility and permeability, with low safety and tolerability.^9^ On the other hand, medicines with solubility in water have been a focus in the pharmaceutical market due to greater bioavailability characteristics, ability to present potency in low doses, and lower costs.^10^ Therefore, evaluating the antidepressant efficacy of new salts is a prospective field of preclinical research due to their potential to improve drug solubility and bioavailability.

At the same time, selenium plays a crucial role as an antioxidant and neuromodulator, with recent studies investigating its relationship with depression.^11,12^ Selenoproteins are critical for bolstering the nervous system’s antioxidant defenses and are also significant for improving mood and supporting the maintenance of metabolic and oxidative functions.^13,14^ Furthermore, the incorporation of selenium into the molecular structure of synthetic compounds results in molecules with neuroprotective properties.^15,16^ A limitation of such molecules lies in their high lipid solubility.

Considering that medicines formulated as salts exhibit greater solubility, bioavailability, and stability, facilitating formulation development and potentially reducing costs,^17,18^ the development of salts of organoselenium compounds could present a promising proposal for creating medicine candidates related to field of selenium research. In this sense, pyridinium salts have attracted attention due to their easy synthesis, chemical reactivity, and significant role as pyridinium ionic liquids, presenting themselves as antimicrobial, anticancer, antimalarial agents, and anticholinesterase inhibitors.^19–21^ Recent studies have reported the synthesis and biological evaluation of selenium-containing pyridinium salts.^22,23^

Therefore, the present study utilized in vitro e in vivo approaches to investigate the antioxidant and monoamine oxidase (MAO) inhibitory effects in the mouse brain, as well as the antidepressant-like effect of selenium-containing pyridinium salts in Swiss mice. Furthermore, the acute toxic class method was employed to predict the safety of salts.

## 2 Results and Discussion

Three selenium-containing pyridinium salts (Figure 1) were selected to verify their potential as antioxidants, MAO inhibitors, and antidepressants in preclinical trials. Because they are relatively soluble in water, the compounds are predicted to have potential in low doses, and oral bioavailability, which is important since most medicines used on a large scale are administered orally.^24^ Poorly water-soluble drugs, with slow drug absorption, lead to inadequate and variable bioavailability and toxicity of the gastrointestinal mucosa.^25,26^ In this context, the ionic nature inherent to pyridinium salts contributes to increasing the solubility of molecules in aqueous media, thus placing these compounds at the forefront of pharmacological investigations. Furthermore, the structural characteristics of the compounds play a crucial role in their attractiveness, as the presence of selenium in their formula is essential for causing antidepressant-like effects.

**Figure 1:**
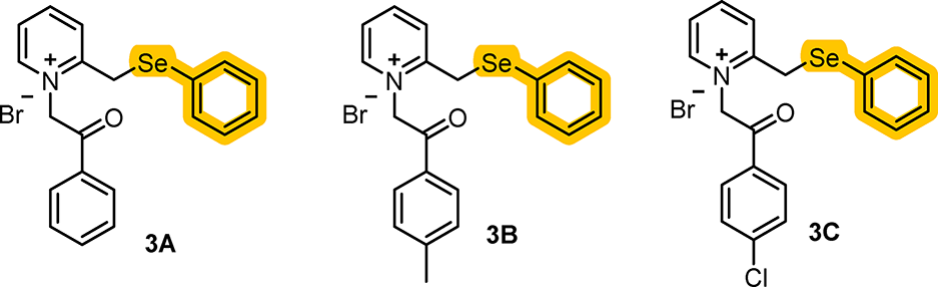
Chemical structures of pyridinium salts 1-(2-oxo-2-phenylethyl)-2-((phenylselanyl)­methyl)­pyridin-1-ium bromide (**3A**), 1-(2-oxo-2- (p-tolyl)­ethyl)-2-((phenylselanyl)­methyl)­pyridin-1-ium bromide (**3B**), and 1-(2-(4-chlorophenyl)-2-oxoethyl)-2-((phenylselanyl)­methyl)­pyridin-1-ium bromide (**3C**).

In vitro tests were performed and demonstrated by protein carbonylation techniques and by the assay of thiobarbituric acid-reactive substances (TBARS) that exhibit antioxidant effects. In addition to these in vitro evaluations, a test was performed to evaluate the activity of brain MAO enzymes, in which all compounds demonstrated efficacy in reducing the activity of MAO-A and MAO-B. Subsequently, in vivo screening was performed, leading to the identification of compound **3B** as the most promising candidate with antidepressant effects. Finally, acute oral toxicity assay was performed, demonstrating that the 50 mg/kg dose was not toxic at biochemical and behavioral levels in female mice.

### 2.1 Salts **3A** , **3B** , and **3C** Inhibit Cerebral MAO-A and MAO-B Activities

MAO enzymes share similar overall architectures, featuring conserved FAD-binding domains and diverse substrate-binding sites, with multiple catalytic mechanisms proposed.^27^ The chemical structures of the studied salts suggest their potential to interact with the MAO active site. Since these salts contain aromatic rings, selenium, and a protonated pyridinium moiety, among other structural features, their inhibition of MAO could involve, at least in part, a combination of hydrophobic, electrostatic, and potentially redox or covalent interactions mediated by selenium. MAO represents a class of metabolizing enzymes present in peripheral and brain tissues. These enzymes use molecular oxygen to catalyze the oxidative deamination of monoamines, producing hydrogen peroxide (H_2_O_2_) and thereby generating significant oxidative stress.^28,29^ Although NAD­(P)H and the electron transport chain are major producers of reactive species, MAO, located in the mitochondrial membrane, can influence the overproduction of reactive species when its activity is increased, leading to problems in the neural tissue.^30^ This underscores the importance of enzyme inhibitors, particularly given that the pathology of depression is linked to the high depletion of neurotransmitters, primarily serotonin, and dopaminergic dysfunction of the corticolimbic system.^29,31,32^

To evaluate the possible inhibition of cerebral MAO-A and MAO-B activities by the Se-based pyridinium salts, analyses were performed at concentrations ranging from 25 to 200 μM. The MAO-A isoform of the enzyme has a high affinity for serotonin deamination. Compounds **3A** and **3C** exhibit significant differences from the control in MAO-A activity, as indicated by one-way ANOVA [*F*_(4, 10)_ = 9.712; *p* = 0.0018 and *F*_(4, 10)_ = 9.115; *p* = 0.0023, respectively]. Dunnett’s post hoc test revealed significant inhibition at concentrations starting at 50 μM for both compounds (Figure 2A,E). Similarly, compound **3B** demonstrated significance in the ANOVA, with *F*_(4, 10)_ = 49.33; *p* < 0.0001, showing reductions of enzyme activity at all concentrations tested (Figure 2C). In addition, MAO-B was analyzed due to its greater affinity for dopamine degradation. Compound **3A** demonstrated effective inhibition of this isoform [*F*_(4, 10)_ = 8.413; *p* = 0.0031]. Dunnett’s post hoc test revealed significant inhibition at concentrations of 50, 100, and 200 μM, as illustrated in Figure 2B. Likewise, compound **3B** demonstrated significance in the ANOVA, with *F*_(4, 10)_ = 12.00; *p* = 0.0008, and Dunnett’s post hoc test revealed inhibition starting at 50 μM (Figure 2D). Finally, compound **3C** [*F*_(4, 10)_ = 23.38; *p* < 0.0001] showed significant inhibition at concentrations of 100 and 200 μM for MAO-B. The IC_50_ values of compounds **3A**, **3B**, and **3C** for MAO-A were 237.7, 73.88, and 77.59 μM, respectively. For MAO-B, the IC_50_ values were 128.9, 91.08, and 125.4 μM for compounds **3A**, **3B**, and **3C**, respectively.

**Figure 2:**
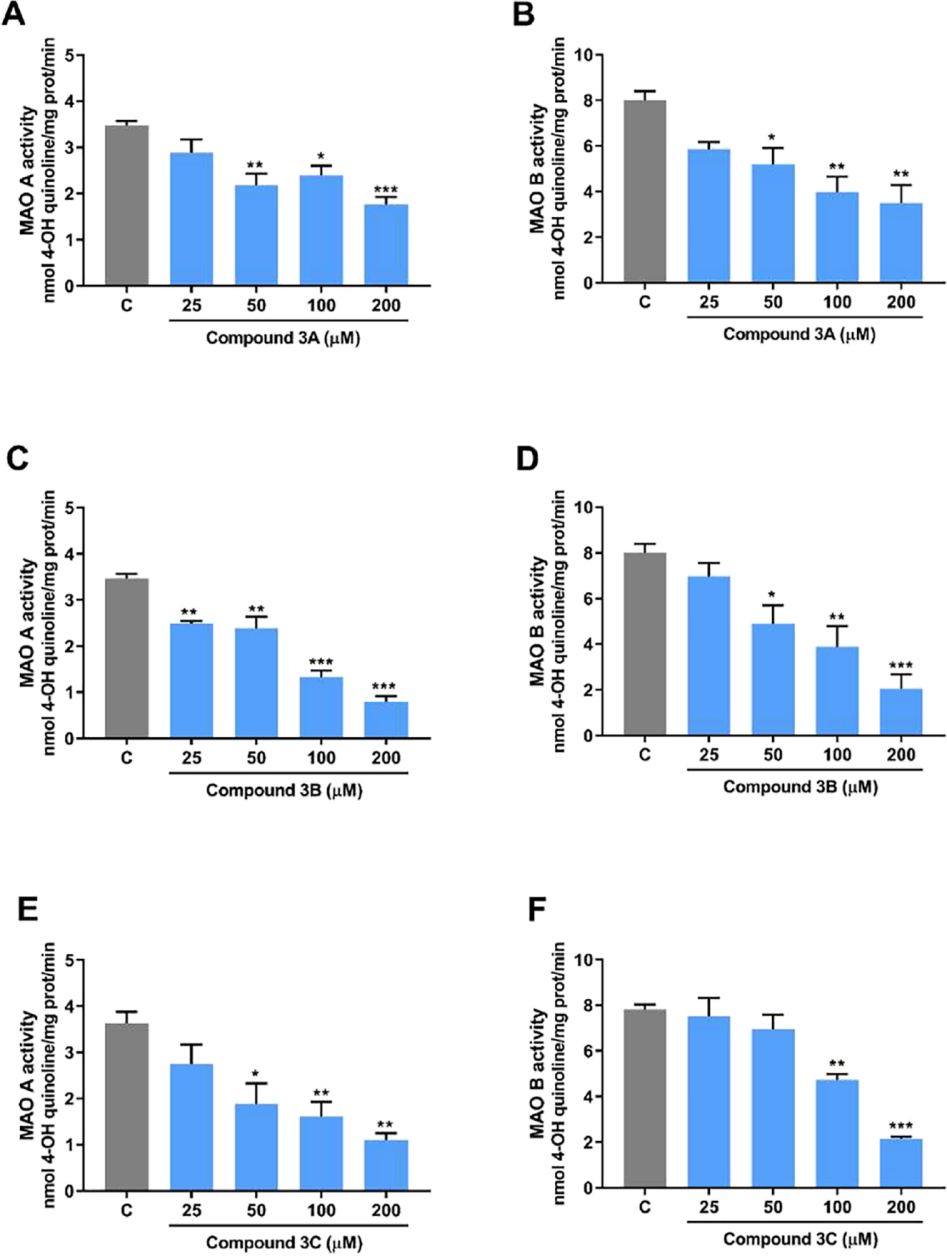
Effects of selenium-containing pyridinium salts (25–500 μM) on the enzyme monoamine oxidase A and B. Effect of compound **3A** on the cerebral activity of MAO-A (A) and MAO-B (B) isoforms; effect of compound **3B** in MAO-A (C) and MAO-B (D), and effect of compound **3C** in MAO-A (E) and MAO-B (F). One-way ANOVA following by Dunnett’s post hoc test was used, and the results were expressed as nmol 4-OH quinoline per milligram of sample protein. *p < 0.05; **p < 0.01; and ***p < 0.001 were used to compare with the control. Abbreviations: C = control group.

The MAO inhibitory activity observed in the compounds is relevant, as current literature advocates combining MAO inhibitors with other antidepressants in patients resistant to conventional depression treatments.^33^ As shown, compound **3B** was the most effective in inhibiting the enzyme, particularly the MAO-A isoform, which has a greater affinity for serotonin. Regarding MAO-B, the isoform for which compound **3B** also had a lower IC_50_, it has a greater affinity for dopamine deamination. Since dysfunction of this enzyme implies oxidative stress and worsening of the depressive condition, this test was performed to guide further studies and to determine the potential effectiveness of starting an in vivo experiment.

The salts exhibited MAO inhibitory activity in the high micromolar range, indicating modest potency when compared to nanomolar-range inhibitors, which could eventually be considered a limitation. Despite this, many pharmacologically relevant compounds can initially display similar profiles and be further optimized for potency, selectivity, and pharmacokinetic properties, and the formation of more active metabolites in vivo cannot be ruled out. Moreover, our study is consistent with previous in vitro studies on other molecules in the micromolar range,^34,35^ while a strong MAO inhibition can be associated with adverse effects (e.g., serotonin toxicity).^36^ It should be emphasized that antidepressant efficacy does not rely solely on MAO inhibition. Multitarget mechanisms, including modulation of monoamine transporters, neurotrophic pathways, or oxidative stress mechanisms,^37^ may compensate for moderate MAO inhibition and result in meaningful antidepressant effects, which remains a question for future investigations. In addition, assays using brain homogenates may reduce apparent potency compared to purified enzymes but offer greater physiological relevance by better reflecting native enzyme behavior. Together, these considerations support the validity of our experimental approach and highlight the potential of these salts, particularly salt **3B**, as promising candidates for antidepressant screening.

### 2.2 Salts **3A**, **3B,** and **3C** Elicit Cerebral Antioxidant Actions

Oxidative stress exacerbates mechanisms implicated in the pathogenesis of depression, including elevated levels of neuroinflammation, imbalanced neurotransmitter signaling, and impaired neurogenesis.^38,39^ In fact, the high activity of the MAO enzyme is a major source of oxidative damage present in pathologies such as depression.^40^ With this in mind, it would be advantageous to work with a compound that presents antioxidant activity. Previous studies have highlighted the antioxidant potential of various selenium species,^13,41^ and compounds containing pyridinium rings.^42^ Therefore, designing hybrids could be a promising strategy for antioxidant activity. In vitro tests were carried out to analyze the antioxidant activity of the salts through TBARS and protein carbonylation assays. It is known that lipid peroxidation is an oxidation process of polyunsaturated fatty acids caused by free radicals. Thus, TBARS are formed as a byproduct of lipid peroxidation and can be detected by the TBARS assay.^43^

The one-way ANOVA yielded significant results for the three compounds **3A**, **3B**, and **3C** (Figure 3) [*F*_(7,16)_ = 20.0; *p* < 0.0001], [*F*_(7,16)_ = 14.76; *p* < 0.0001], and [*F*_(7,16)_ = 12.71; *p* < 0.0001], respectively, in the TBARS assay. The Tukey post hoc test showed that sodium nitroprusside (SNP) induced oxidative damage in all experiments. For compounds **3A** and **3B**, concentrations of 100, 200, and 500 μM were effective in reversing the oxidative damage caused by the SNP inducer. Regarding compound **3C**, the effective concentrations were 50–500 μM. Additionally, the 50% inhibitory concentration (IC_50_) was calculated, and values of 150, 130.2, and 101.8 μM were obtained for compounds **3A**, **3B**, and **3C**, respectively. Trolox, a standard antioxidant used as positive control, effectively reversed oxidative damage in all experiments, as expected (*p* < 0.0001, *p* = 0.0002, and *p* = 0.0004, respectively).

**Figure 3:**
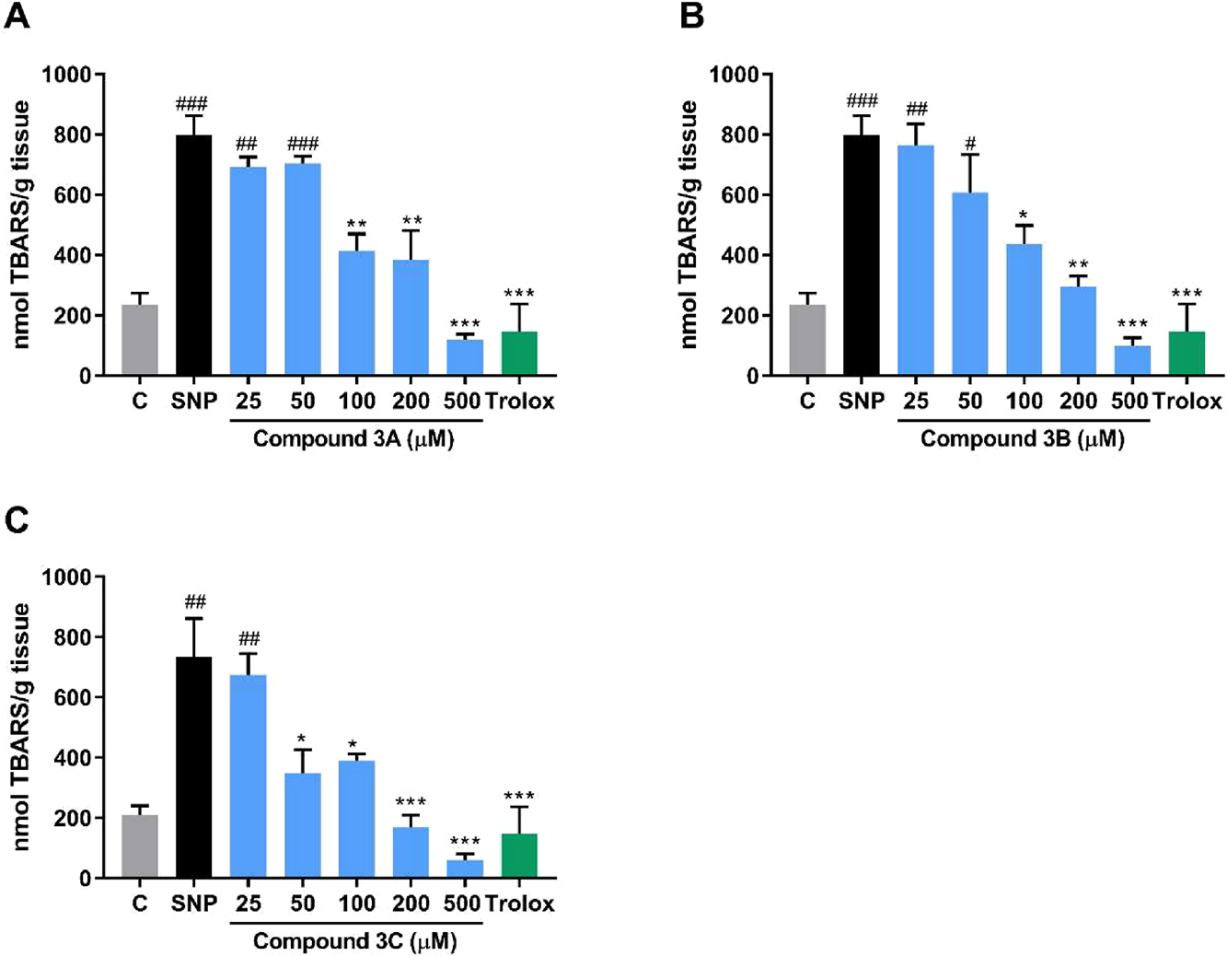
Effects of selenium-containing pyridinium salts (5–500 μM) on cerebral lipid peroxidation level (TBARS). (A) Compound **3A**, (B) compound **3B**, (C) compound **3C**. Three independent experiments (*n* = 3) were performed in duplicate. Trolox was used as positive control and tested at a concentration of 100 μM. A one-way ANOVA followed by Tukey’s post hoc test was applied, and results were expressed as nmol of TBARS per gram of tissue. ^#^ *p* < 0.05, ^##^ *p* < 0.01, and ^###^ *p* < 0.001 compared with the control. *p < 0.05, **p < 0.01, and ***p < 0.001 in relation to the SNP group. Abbreviations: C = control; SNP = sodium nitroprusside.

Furthermore, a parallel TBARS experiment was conducted using a standard alkyl aryl selenide (2-((phenylselanyl)­methyl)­pyridine) to assess whether the redox activity of the salts depends solely on the presence of selenium or is also influenced by other structural features. We observed a loss of antioxidant potency, as significant effects were detected only at 500 μM for this standard (Figure S1). This suggests that, although both selenium^13^ and pyridine derivatives^44^ have been reported to possess antioxidant properties, the activity observed in the current compounds likely depends on the contribution of the pyridinium form (protonated nitrogen) and/or other molecular components. While pyridinium is less capable of donating electrons than pyridine, it may modulate antioxidant activity through electrostatic interactions and improved solubility, enhancing biological effects in aqueous media (e.g., ROS-producing enzymes also could serve as potential targets). In other words, the overall molecular assembly is required to achieve the most effective antioxidant activity.

As reported by Castro et al., the antioxidant mechanisms of these salts can be attributed to electron transfer (ET) and hydrogen atom transfer (HAT), as well as to their glutathione S-transferase (GST)-mimetic activity, an enzyme involved in antioxidant defense and detoxification.^22^ Moreover, additional mechanisms cannot be excluded, such as the modulation of ROS-producing enzymes, including MAO (as demonstrated here), NADPH oxidases, xanthine oxidase, nitric oxide synthases, among others.

The present data reveal that all salts have antioxidant action, with the potential to protect brain tissue against lipid peroxidation. Furthermore, findings indicate compound **3C** as the most effective. Our findings are corroborated by data in the literature, which demonstrate the antioxidant effectiveness of pyridinium^42^ and selenium^41^ compounds in brain tissue. However, it is worth mentioning that the present compounds, unlike those mentioned above, are soluble in water.

Also, proteins are oxidatively modified by various reactive species, including reactive oxygen species and lipid peroxidation derivatives. Protein carbonylation is a main hallmark of oxidative damage to proteins.^45^ To complement our studies, the protein carbonylation assay was carried out with the three compounds (25–200 μM) (Figure 4A-C). A considerable increase in protein carbonyl content is associated with the development of neurodegenerative diseases.^46^

**Figure 4:**
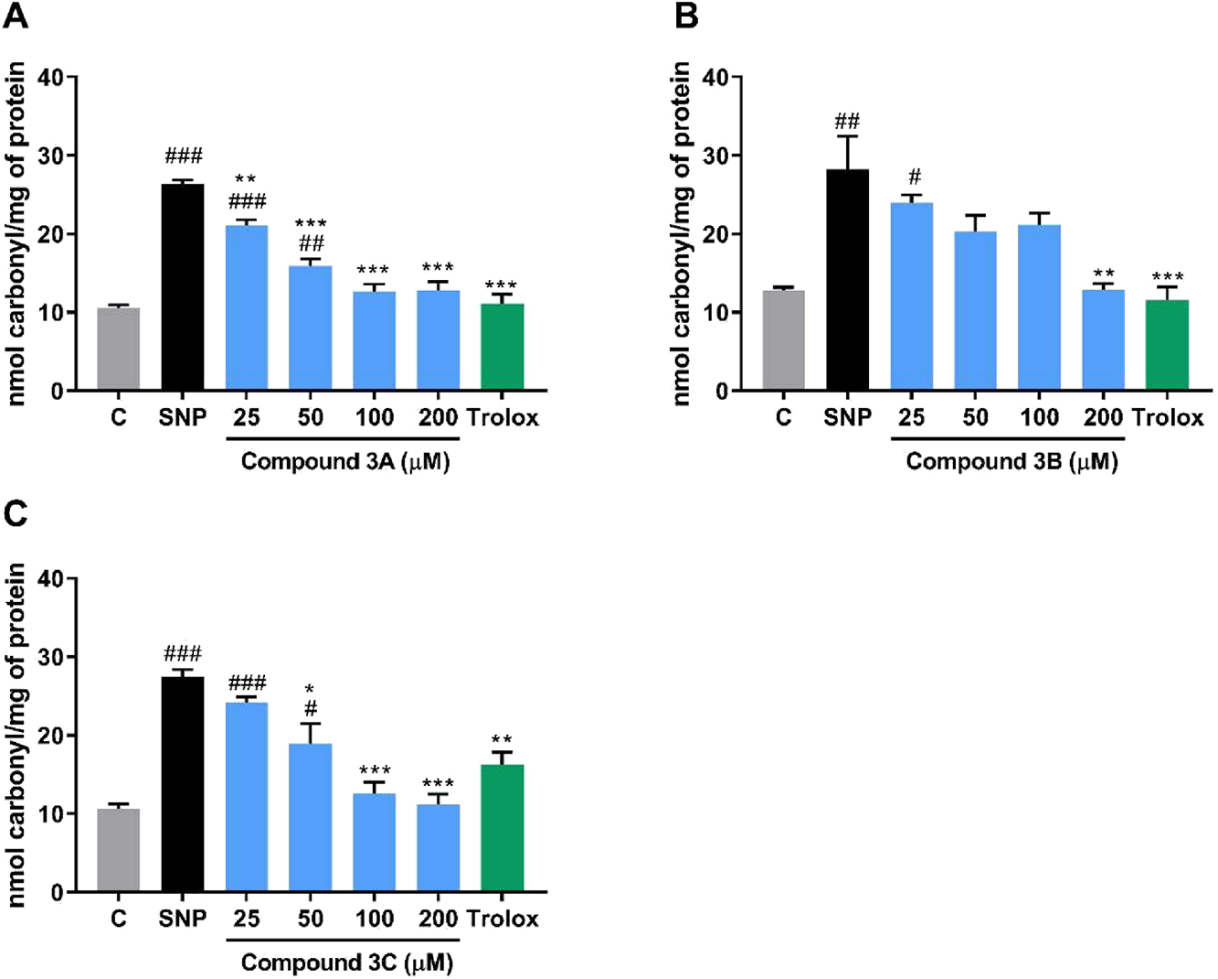
Effects of selenium-containing pyridinium salts (25–500 μM) on protein carbonylation. (A) Compound **3A**, (B) compound **3B**, (C) compound **3C.** Three independent experiments (*n* = 3) were performed in duplicate. Trolox (100 μM) was used as a positive control. A one-way ANOVA followed by Tukey’s post hoc test was used, and results were expressed as nmol of carbonyl per gram of tissue. ^#^ *p* < 0.05, ^##^ *p* < 0.01 and ^###^ *p* < 0.001 were used for comparison with the control. p*<0.05, p**p < 0.01 and ***p < 0.001 in relation to the SNP group. Abbreviations: C = control; SNP = sodium nitroprusside.

In the in vitro test, one-way ANOVA showed significance for all salts, with statistical values of *F*_(6, 14)_ = 47.06; *p* < 0.0001 for **3A**, *F*_(6, 14)_ = 9.848; *p* = 0.0002 for **3B**, and *F*_(6, 14)_ = 20.11; *p* < 0.0001 for **3C**. For compound **3A**, post hoc analysis revealed that concentrations of 25 and 50 μM were significantly different from the control group. However, all concentrations could attenuate the formation of protein carbonylation induced by SNP. Compound **3B** was significantly different from the control group at the concentration of 25 μM and was effective in reversing protein damage at 200 μM. Finally, compound **3C** was significantly different from the control group at concentrations of 25 and 50 μM and effective in reducing protein carbonylation at concentrations of 50, 100, and 200 μM. In all three assays, Trolox (100 μM) was used as a positive control, proving to be effective in validating the experiment. Regarding the IC_50_ of compounds **3A, 3B**, and **3C**, values obtained were 130.1, 229.8, and 110.8 μM, respectively.

The three compounds under investigation were effective as antioxidant agents in vitro, reducing protein carbonylation and lipid peroxidation in mouse brain tissue. In addition, they were effective in reducing oxidative damage in the liver.^22^

### 2.3 In Silico Test for Prediction of Absorption, Distribution, Metabolism, Excretion, and Toxicity (ADMET)

The pkCSM Web site predicts small-molecule pharmacokinetic properties using graph-based signatures.^47^ This in silico analysis was conducted to gain insights into how the compounds might perform in in vivo experiments. It is important to note that these predictions are speculative and may or may not reflect the actual behavior in the human body. The results are detailed in the Supporting Information, with Tables S1, S2, and S3 representing compounds **3A**, **3B**, and **3C**, respectively.

We highlight five of the various parameters analyzed. All compounds demonstrated high values for intestinal absorption (73.435, 73.538, and 77.434, respectively), exceeding the acceptable threshold of 30. Additionally, all three compounds exhibited permeability properties in the central nervous system, with logP values above the recommended threshold of −2. The logP values for the compounds were −1.209, −1.262, and −1.209, respectively.

The median lethal dose values for the three compounds were all above 1000 mg/kg, and none of them exhibited hepatotoxicity. Furthermore, all three compounds showed solubility values with log mol/L greater than −2, indicating that they are water-soluble.

### 2.4 Acute Oral Toxicity Protocol

Safety protocols use relatively high doses to detect potential toxic effects in animals. In the present study, an acute oral toxicity design was performed to evaluate the salts **3A** and **3B** (50 and 300 mg/kg) following the guidelines outlined in Protocol 423–Acute Toxic Class Method of the Organization for Economic Cooperation and Development (OECD).^48^ There were no instances of animal mortality associated with compound treatments, and evaluations of physical signs did not indicate any observable changes. Compound **3C** was not evaluated here because it has already been tested with the same methodology layout, and there were no signs of toxicity in the animals.^22^

During toxicity testing, monitoring food and water intake along with body weight helps assess the impact of substances on animal health and well-being and can predict potential adverse effects. In our study, food and water intake assessments were conducted every 3 days, resulting in 5 days of data collection. It was seen that the groups that received compounds **3A** and **3B** ate similar to the control group, at doses of 50 mg/kg [*F*_(2, 12)_ = 2.761; *p* = 0.1032] or 300 mg/kg [*p* = 0.8470; Kruskal–Wallis test] (Figure 5A).

**Figure 5:**
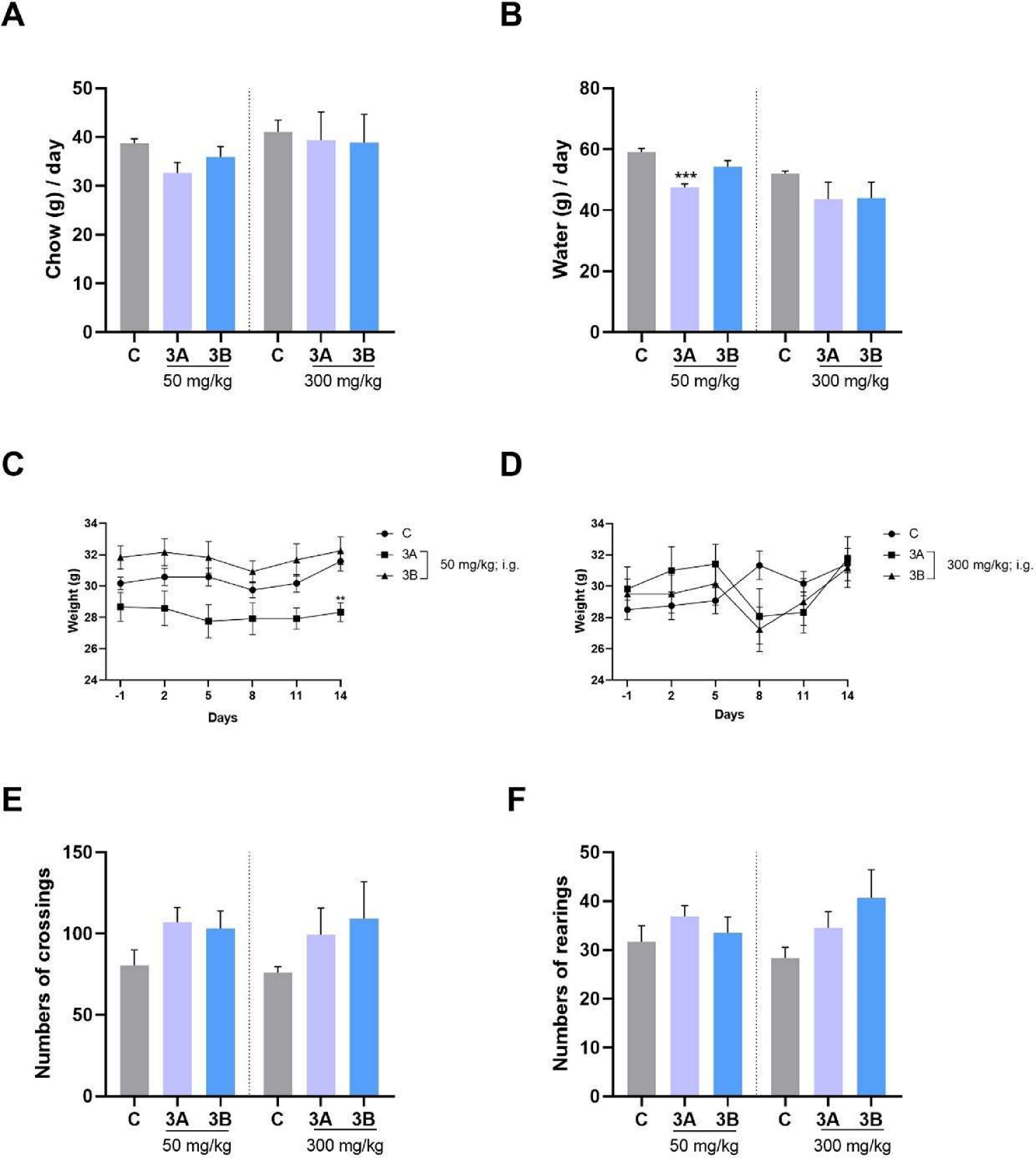
Measurement of intake, body weight and locomotion after administration of compounds **3A** and **3B** at high doses (50 and 300 mg/kg). Food intake (A), water intake (B), body weight (50 mg/kg) (C), body weight (300 mg/kg) (D), crossings in OFT (E) and rearings in OFT (F). The average intake of the cage containing 3 animals was taken every 3 days within the protocol interval (*n* = 5 cages) for graphs A and B. For images C and D, data related to each animal (*n* = 6) were used. Values are expressed as mean ± SEM. One-way or two-way ANOVA followed by Dunnett’s or Bonferroni’s post hoc test was used. ***p < 0.001 was used for comparison to the control. Abbreviations: C = control.

This suggests that the treatment did not significantly affect appetite or food consumption. Notably, water consumption decreased only in the group receiving compound **3A** at the 50 mg/kg dose, but not in the group receiving compound **3B** [*F*_(2, 12)_ = 14.85; *p* = 0.0006]. However, when comparing the water consumption of the groups that received compounds **3A** and **3B** at a dose of 300 mg/kg with the control, there was no significant difference [*p* = 0.1503; Kruskal–Wallis test] (Figure 5B).

When body weight gain (14 days) was evaluated, it was similar across all groups [50 mg/kg, *F*_(2, 15)_ = 1.702; *p* = 0.2156; and 300 mg/kg, *F*_(2, 15)_ = 1.624; *p* = 0.2300]. A reduction in body weight of the animals was observed at the last measurement (last day) in group **3A** at a dose of 50 mg/kg (but not for **3B**) compared with the control. The group treated with compound **3B** did not differ significantly [*F*_(10, 75)_ = 0.4947; *p* = 0.8884]. For the dose of 300 mg/kg of both compounds, although interaction had been detected [*F*_(10, 75)_ = 6.186; *p* < 0.0001], no significant difference was observed in relation to the control as revealed by post hoc (*p* > 0.05) (Figure 5C,D).

Additionally, the open field test (OFT) was performed. The OFT is validated to assess whether a drug will have psychotropic effects, such as psychostimulant actions (increasing movements on the device) or sedative actions (showing reduced locomotion) as well as reduced motor performance by physiological discomfort/toxicity.^49,50^

In this sense, the animals did not exhibit changes in locomotor (Figure 5E) or exploratory behavior (Figure 5F) at any tested dose of the two compounds under investigation ([*F*_(2,15)_ = 2.146; *p* = 0.1515] and [*F*_(2,15)_ = 0.7780; *p* = 0.4770], respectively for crossing and rearing at a dose of 50 mg/kg; and [*p* = 0.4402; Kruskal–Wallis test] and [*F*_(2,15)_ = 2.322; *p* = 0.1323], respectively, for crossing and rearing at dose 300 mg/kg. These findings indicate no adverse effects of the treatment on spontaneous locomotor performance of animals.

Additionally, the results regarding the activity of the enzymes alanine aminotransferase (ALT) (Figure 6A) and aspartate aminotransferase (AST) (Figure 6B), as well as the levels of urea (Figure 6C) in the plasma of animals treated with compounds **3A** and **3B**, at doses of 50 and 300 mg/kg, are presented. One-way ANOVA shows that ALT activity remained unchanged with any of the doses administered of the salts [*F*_(2, 15)_ = 0.8442; *p* = 0.4493 and *F*_(2, 15)_ = 0.3333; *p* = 0.7217]. The findings suggest that treatment with a dose of 50 mg/kg did not affect AST activity [*F*_(2, 15)_ = 0.4535; *p* = 0.6439], while the post hoc Dunnett shows an increase was observed at a dose of 300 mg/kg of compound **3B** [*F*_(2, 15)_ = 4.822; *p* = 0.241]. Urea levels also remained similar in comparison of compound **3A** and **3B** groups relative to control at both doses [*F*_(2, 15)_ = 1.118; *p* = 0.3528; *F*_(2, 15)_= 2.973; *p* = 0.818].

**Figure 6:**
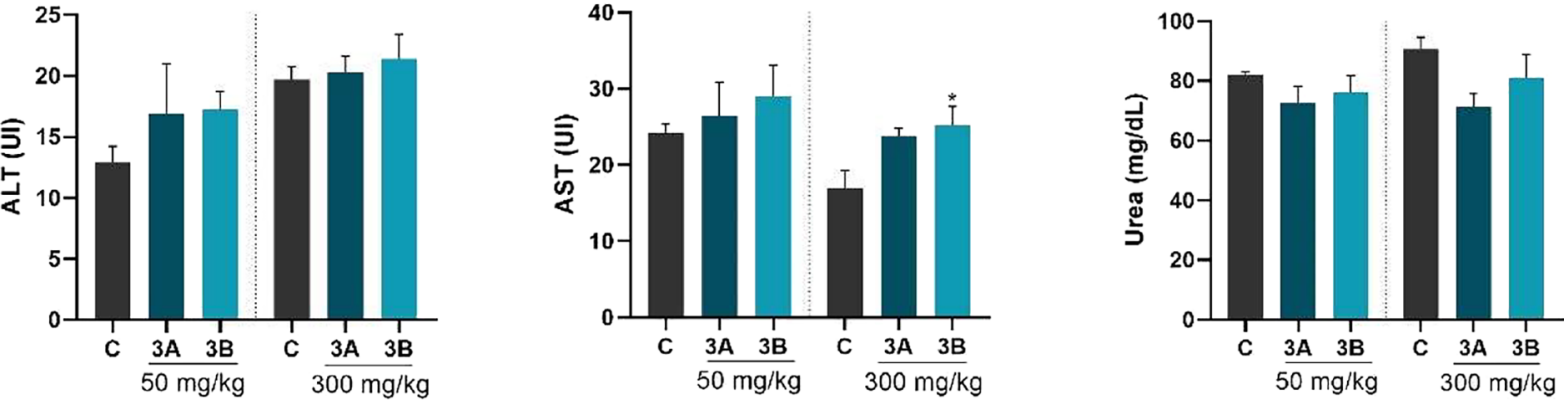
Ex vivo assays were conducted to evaluate the acute oral toxicity of compounds **3A** and **3B** at doses of 50 and 300 mg/kg, i.g. in female mice. Plasma AST (A), ALT (B) activities, and urea levels (C). One-way ANOVA with Dunnett’s post hoc test, when necessary, was applied to determine statistical significance (*n* = 6/group). Values are presented as mean ± SEM *p < 0.05 compared with the control group. *p < 0.05 was used for comparison with the control. C = control.

Ex vivo lipid peroxidation assays were conducted in the brain, liver, and kidneys for both compounds (Figure S5). The evaluations indicated that doses of 50 and 300 mg/kg did not induce oxidative damage in the brain and/or kidneys (Figure S5A–C). On the other hand, in relation to the liver, an increase in damage was observed only at the 50 mg/kg dose of compound **3B**, which was not evident at the higher dose. Furthermore, in the kidney there was no oxidative damage at any of the doses, of both compounds, as shown in Figure S5B.

Although there was an increase in oxidative damage, the doses tested were 50 times higher than the effective dose observed in vivo. The toxicity value of compounds derived from pyridinium salts appears favorable compared with established antidepressants, such as fluoxetine, a widely used medicine, which has been shown to have a median lethal dose (LD_50_) of 248 mg/kg.^51^ Considering the absence of physical signs of toxicity, such as hair erection, ptosis, convulsions, changes in feeding, weight changes or mortality in animals,^52^ compound **3B** appears promising for further investigation.

### 2.5 In Vivo Antidepressant-like Activity and Absence of Locomotor Impairments

#### 2.5.1 Compound **3B** Elicits Antidepressant-like Actions in the Mouse Tail Suspension Test (TST)

Based on preliminary results and the expected modulation of the nervous system by compounds **3A–C**, the tail suspension test (TST) was used to evaluate the antidepressant-like effects of these compounds in male Swiss mice.

It was demonstrated that the TST and forced swim test (FST) allow observation of a significant reduction in immobility with just a single injection of antidepressant drugs.^53^ This permits the tests to be used to rapidly screen new compounds for their antidepressant-like activity. When comparing the two recommended tests, the TST causes less animal suffering and stress, which is why it was chosen for screening our compounds.^54^ In addition, there is a greater sensitivity of the TST with drugs that act on the serotonergic pathway.^55^ A fixed dose of 5 mg/kg and a 30 min pretreatment time were chosen for response analysis from the TST. Four minutes before this test, the animals underwent the OFT. (Figure 7A). In the TST, one-way ANOVA revealed significance for both parameters: latency time [*F*_(4, 36)_ = 10.42; *p* < 0.0001] (Figure 7B) and total immobility time [*F*_(4,36)_ = 7.194; *p* = 0.0002] (Figure 7C). Dunnett’s post hoc indicated that compound **3B** and the positive control (fluoxetine at a dose of 20 mg/kg) significantly increased latency time, i.e., they took longer to exhibit despair-like behaviors. Regarding the total immobility parameter, the findings were consistent with latency results, showing shorter total immobility times, indicative of reduced depressive-like behavior in mice treated with compound **3B** and fluoxetine. The Cohen-D test was performed, and it was concluded that the effect is high (*d* > 1.77). Taken together, these findings indicate that **3B** can effectively reduce behaviors associated with despair and, therefore, may have therapeutic potential as an antidepressant.

**Figure 7:**
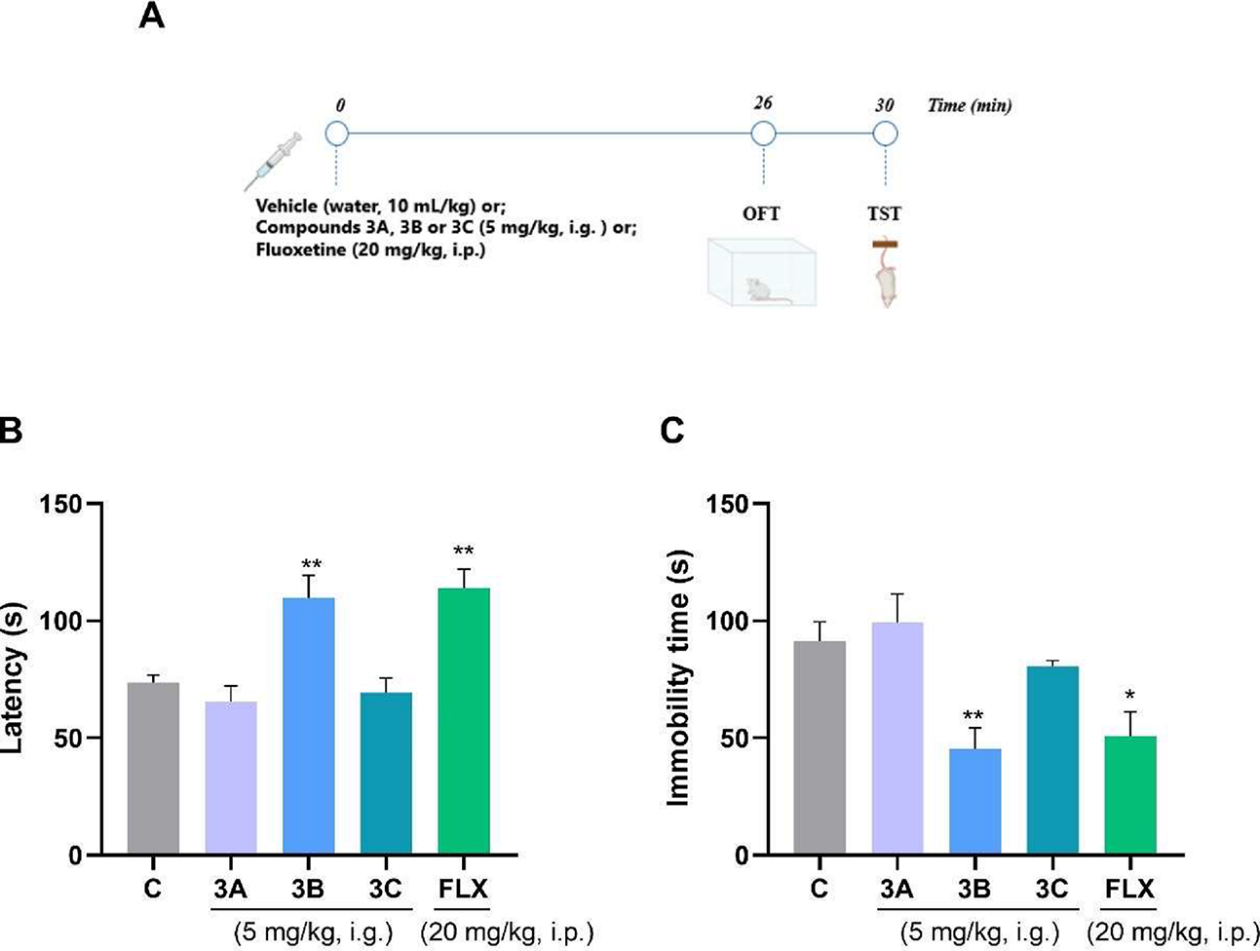
Effects of compounds **3A**, **3B**, and **3C** on the mouse TST (*n* = 7–9 mice per group). Experimental design (A). Latency to the first immobility episode (s), (B) and total immobility time (s) (C). Fluoxetine at a dose of 20 mg/kg (i.p.) was used as a positive control. One-way ANOVA followed by Dunnett’s post hoc test was used. *p < 0.05; **p < 0.01; and ***p < 0.001 were used to compare with the control. Abbreviations: C = control; FLX = fluoxetine.

One-way ANOVA revealed no significant difference in the OFT for the number of crossings [*F*_(4,36)_ = 1.185; *p* = 0.3340] or rearings [*F*_(4,36)_ = 0.5614; *p* = 0.6922] (Figure S2). These results show no signs of toxicity or psychostimulant effects and confirm the reliability of the TST data, as there was no mobility changes.

Therefore, it was concluded that compound **3B** was effective in producing antidepressant-like effects in male Swiss mice. To determine the effective dose and duration of action of compound **3B**, time- and dose–response curves were performed using the TST, a widely used test.^56,57^

#### 2.5.2 Compound **3B** Induces Rapid Antidepressant-like Effects in the Mouse TST

After identifying compound **3B** as having antidepressant-like properties in the initial screening, a time-response curve was constructed. The tested time points included 15, 30, 60, and 120 min (Figure 8A). ANOVA revealed a statistically significant difference in the two parameters analyzed in the TST: latency time [*F*_(4, 41)_ = 8.729; *p* < 0.0001] and total immobility [*F*_(4, 41)_ = 11.10; *p* < 0.0001] (Figure 8B,C). Groups tested at 15 and 30 min exhibited increased latency time. Concerning immobility, Dunnett’s post hoc test demonstrated significance in the 15, 30, and 60 min groups compared with the control.

**Figure 8:**
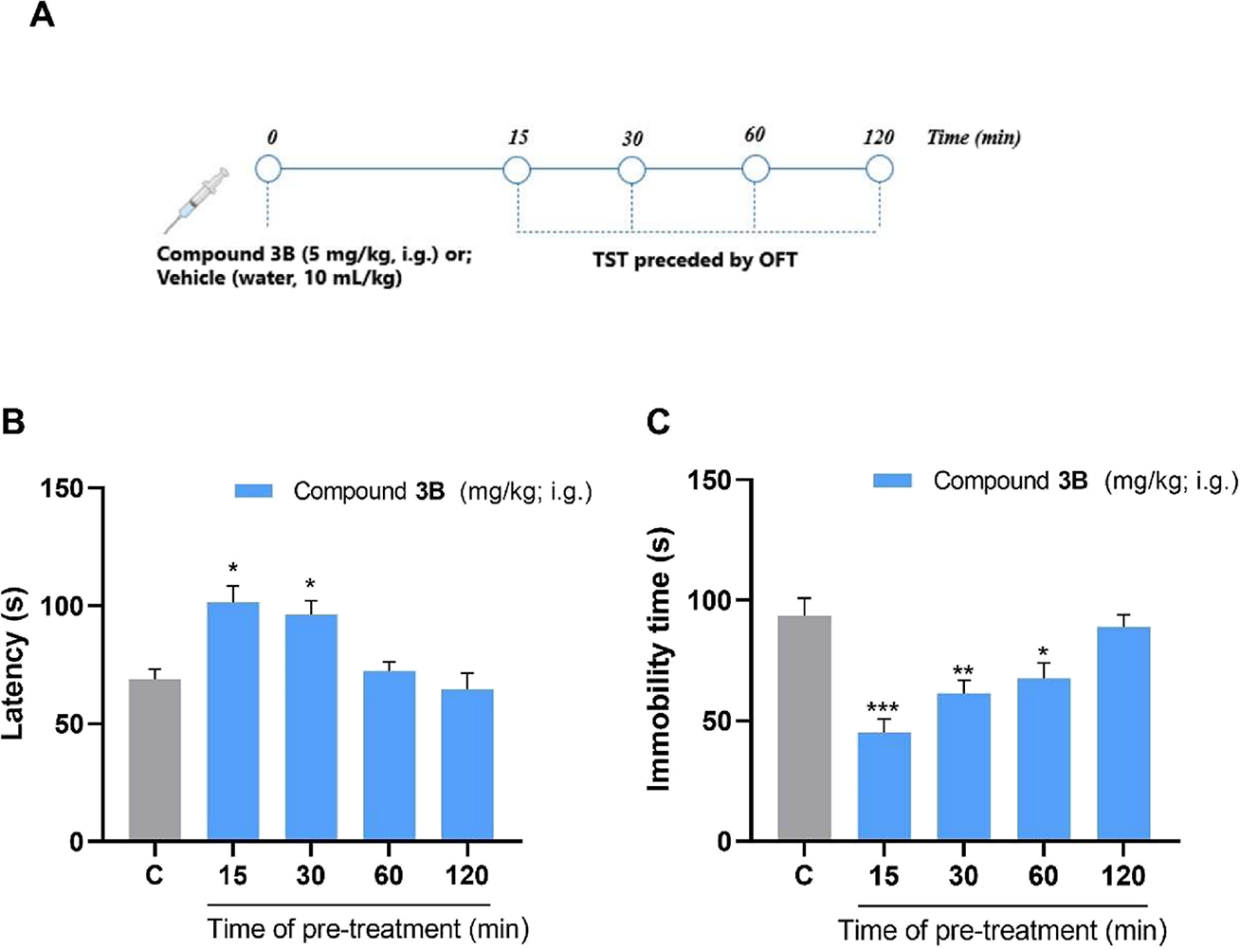
Result of the time-response curve of compound **3B** (5 mg/kg, i.g.) at times of 15–120 min in the TST (*n* = 8–10 mice per group). Experimental design (A). Latency to the first immobility episode (s) (B); and the total time of immobility (s) (C). One-way ANOVA followed by Dunnett’s post hoc test was used. *p < 0.05; **p < 0.01; and ***p < 0.001 were used for comparison with the control. Abbreviations: C = control.

One-way ANOVA revealed no significant difference in OFT conducted during the time-response curve experiment, in relation to the number of crossings [*F*_(4, 41)_ = 11.10; *p* = 0.163] or rearings [*F*_(4, 41)_ = 1.716; *p* = 0.1649] (Figure S3), indicating that the treatment with compound **3B** did not affect the locomotor and exploratory activities of mice at any time point.

Indeed, time points ranging from 15 to 30 min proved significant for the antidepressant action. A duration of 30 min was chosen for the continued protocol, as most studied compounds containing selenium exhibit this duration of action.^15,58^ Fluoxetine, a selective serotonin reuptake inhibitor, was used as a positive control to validate the antidepressant-like effect of the studied compound. Similar to other compounds, fluoxetine has an antidepressant-like action time of 30 min.^59^ Unlike compound **3B**, a decrease in rearings was observed in the OFT in animals that received fluoxetine in this experimental set, a phenomenon also noted in other studies.^60,61^ Antidepressants without psychomotor stimulant properties may show a significant reduction in exploratory activity. Fluoxetine (30 mg/kg), dothiepin (10 mg/kg), and venlafaxine (50 mg/kg) produced 89, 73, and 62% reductions in rodent locomotor activity, respectively.^61^

#### 2.5.3 Compound **3B** Demonstrates Antidepressant-like Activity at Low Doses

The oral route is the most common and preferred method for drug delivery due to its safety, efficiency, noninvasiveness, and minimal discomfort to patients. Despite these advantages, the development of oral formulations presents several challenges mainly due to drugs’ physicochemical properties, including poor water solubility.^62^ In preclinical research within the field of organic selenium compounds, these lipophilic compounds often require oily vehicles, such as canola oil.^58,63^ Nevertheless, it is conjectured that lipophilic compounds may encounter challenges regarding dissolution in gastrointestinal fluids and subsequent absorption. In this context, Prigol and colleagues found that diphenyl diselenide (PhSe)_2_, an organoselenium compound, exhibits poor solubility in isotonic phosphate buffer, and even at high doses (500 mg/kg dissolved in oil, per oral), it yields very low levels of selenium in plasma and predominantly accumulates in fatty tissues. These reports suggest that the limited solubility in gastrointestinal fluid may hinder (PhSe)_2_ absorption into the bloodstream, posing challenges for oral formulation.^64–66^

Unlike most organoselenium compounds, compound **3B** stands out for its relative solubility in water. Given its chemical characteristics as a salt and to obtain a more comprehensive efficacy profile, we extended our experiments to test lower doses. Therefore, after establishing a 30 min time frame for the compound to elicit an antidepressant-like effect, a dose–response curve (0.5–5 mg/kg) was outlined, with fluoxetine as a positive control (Figure 9A).

**Figure 9:**
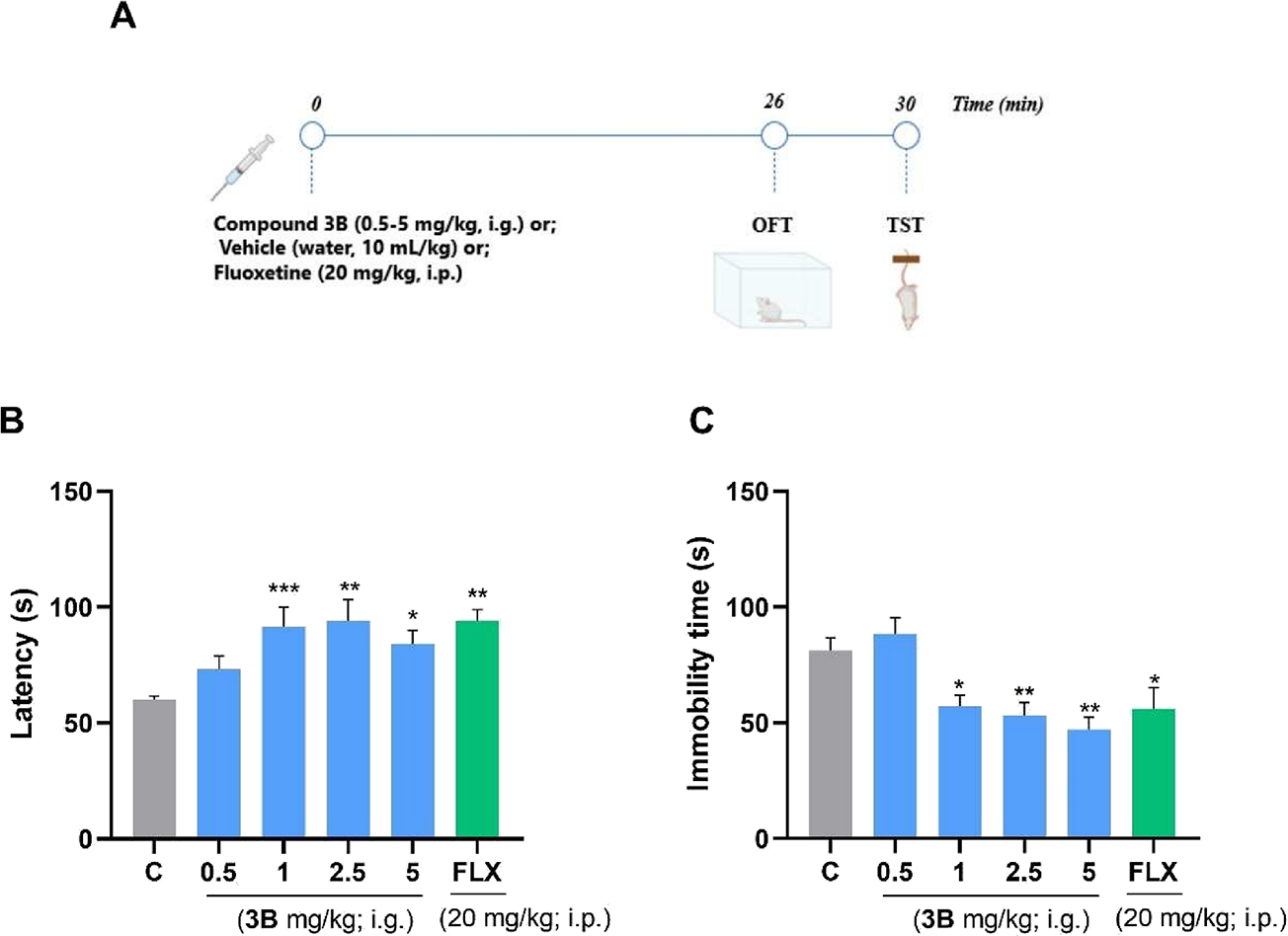
Dose–response curve of compound **3B** (0.5–5 mg/kg, i.g.) in the mouse TST. (A) Experimental design. (B) Latency to the first immobility episode (s), and (C) the total immobility time (s). Fluoxetine (20 mg/kg, i.p.) was used as a positive control (*n* = 8–11 mice per group). One-way ANOVA followed by Dunnett’s post hoc test was used. *p < 0.05; **p < 0.01; and ***p < 0.001 were used to compare with the control. Abbreviations: C = control; FLX = fluoxetine.

In the TST, ANOVA revealed a significant difference for both latency time [*F*_(5, 54)_ = 5.469; *p* = 0.0004] and total immobility time [*F*_(5, 54)_ = 5.402; *p* = 0.0004] (Figure 9B,C). Latency time increased in mice receiving doses of 1, 2.5, and 5 mg/kg of the compound **3B**, as also observed in the fluoxetine group. Regarding total immobility time, doses of 1, 2.5, and 5 mg/kg were significantly reduced compared with the control group. Fluoxetine effectively reduced the immobility time of the animals. The decrease in immobility time and the increase in latency indicate antidepressant-like behavior.

In the OFT (Figure S4), no changes were observed in the number of crossings, suggesting that the animal’s locomotor capacity was not affected by any of the doses tested [*F*_(5,54)_ = 0.4639; *p* = 0.8014]. Similarly, the different doses of compound **3B** did not impact on the exploratory behavior profile of the animals, as can be seen by the number of rearings. However, the fluoxetine group exhibited decreased rearings compared with the control group [*F*_(5,54)_ = 7.331; *p* < 0.0001].

This data set revealed that compound **3B** exhibits efficacy at relatively low doses, with the minimum effective dose for antidepressant-like action being 1 mg/kg. These salts give rise to other related molecules that have been previously studied, such as 2-phenyl-1-(phenylselanyl)­indolizine, which was effective from a dose of 10 mg/kg.^67^ Differences in chemical properties, such as lower water solubility of this related compound, likely account for the observed variations in potency. The moderate water solubility of compound **3B** may be a key factor underlying its efficacy at lower doses. Similar to our findings, water-soluble salts based on benzofuroxan derivatives have been shown to have antileukemic activity after single administration at doses of 1.25–5 mg/kg.^68^ A water-soluble compound composed of salt has been studied that has been shown to be useful in mitigating the inflammatory consequences in neuroinflammatory and neurodegenerative diseases.^69^ Solubility is the main parameter required to achieve the desired concentration of the drug in the systemic circulation and achieve the desired pharmacological effect, in addition to presenting brain permeability.^70^ Drugs with low water solubility generally require high doses to achieve therapeutic plasma concentrations after oral administration.^71^

Here, compound **3B** exhibited therapeutic action at low doses, which could promote its pharmacological utility as an antidepressant compared with numerous other organoselenium compounds, thereby decreasing the likelihood of adverse effects. Besides, in silico ADMET predictions and acute oral toxicity assays revealed relative safety of salt **3B**. Although MAO inhibition and antioxidant mechanisms could contribute to its antidepressant-like effects, we also do not rule out other mechanisms, as many relevant compounds have demonstrated multitarget actions.

## 3 Material and Methods

### 3.1 Chemicals

Compounds 1-(2-oxo-2-phenylethyl)-2-((phenylselanyl)­methyl)­pyridin-1-ium bromide (**3A**), 1-(2-oxo-2-(*p*-tolyl)­ethyl)-2-((phenylselanyl)­methyl)­pyridin-1-ium bromide (**3B**), and 1-(2-(4-chlorophenyl)-2-oxoethyl)-2-((phenylselanyl)­methyl)­pyridin-1-ium bromide (**3C**) (Figure 1) were synthesized in the Clean Organic Synthesis Laboratory, from the Federal University of Pelotas (LASOL, UFPel).^23^

Compounds **3A-C** were prepared by the following procedure^23^: In a round-bottomed flask were added 2-(methylchalcogenyl)-pyridine **1** (1.0 mmol), 2-bromoacetophenone derivative **2** (1.2 mmol) and ethyl acetate (5.0 mL) as the solvent. The resulting solution was vigorously stirred at 70 °C (oil bath), to form a white precipitate. After 24 h, the precipitate was filtered off and washed with ethyl acetate (3 × 2 mL) to remove unreacted starting materials and other soluble impurities. The solvent was removed under reduced pressure. This procedure allowed the synthesis of these new compounds in high purity, and in 62–67% yield (see SI for pictures of NMR spectra). The chemical structures of the compounds were confirmed by nuclear magnetic resonance techniques for carbon-13 and hydrogen (^13^C and ^1^H NMR). Spectral data and pictures of the NMR spectra are presented in the Supporting Information (Pages S12–S17).

All three compounds are relatively soluble in water. The compound at a dose of 50 mg/kg was diluted in water using a magnetic stirrer. Fluoxetine was purchased from EMS Pharmaceuticals in Brazil and diluted in saline for use as a positive control. All other chemicals were obtained from Sigma (St. Louis, MO, USA) or other standard commercial suppliers. For in vivo experiments, substances were administered to animals at a constant volume of 10 mL/kg body weight (v/w).

#### 3.1.1 Animals

Adult male and female Swiss mice, weighing between 25 and 30 g, were obtained from a local colony at the Central Animal Facility at the Federal University of Pelotas, Brazil. The mice were maintained under standard conditions: a temperature of 22 ± 2 °C, with pelleted food and tap water provided ad libitum on a 12-h light-dark cycle, with the light on at 7:00 a.m. All experiments were approved by the animal experimentation committee (027542/2021–11 and 001363/2022-34) affiliated with the National Council for the Control of Animal Experimentation (CONCEA) and conducted in compliance with the National Institutes of Health Guide for the Care and Use of Laboratory Animals. We implemented all necessary measures to ensure that the studies strictly adhered to animal welfare standards and ethical guidelines. All behavioral tests were performed during the light cycle (9 a.m. to 1 p.m.).

### 3.2 In Silico

#### 3.2.1 Pharmacokinetic Studies

Pharmacokinetic properties were evaluated in silico using the online platform “pkCSM-Pharmacokinetics”, developed by Accelrys Software, Inc. in San Diego, CA, United States. The analyzed components included analysis, distribution, metabolism, excretion, and toxicity (ADMET). The method uses graph-based signatures to develop predictive models to evaluate each of these periods.^47^ To evaluate compounds **3A**, **3B**, and **3C**, their 2D structures were designed using ChemDraw Ultra 12 software and then converted to SMILES format. This format was subsequently uploaded to the online pkCSM server (https://biosig.lab.uq.edu.au/pkcsm/) to obtain the predictive results for ADMET.^72^

### 3.3 In Vitro Assays

Brains from mice of both sexes were used for in vitro assays, each performed in duplicate across three independent experiments (*n* = 3), conducted on different days.

#### 3.3.1 TBARS

The brain tissues were homogenized in a 50 mM Tris-HCl buffer at pH 7.4, using a 1:10 ratio (w/v). The freshly prepared homogenate was then centrifuged at 900*g* for 10 min, yielding a low-speed supernatant (S_1_). Lipid peroxidation was evaluated using the TBARS method as previously described.^73^ Lipid peroxidation was induced by 0.3 mM SNP. A 100 μL aliquot of S_1_ was incubated for 1 h at 37 °C in the presence of the compounds **3A**, **3B**, and **3C** at concentrations ranging from 25 to 500 μM, and SNP or water (to control). The color reaction was initiated by adding 500 μL of a 0.8% thiobarbituric acid (TBA) solution, followed by 200 μL of 8.1% sodium dodecyl sulfate (SDS) and 500 μL of acetic acid (pH 3.4). The reaction mixture was incubated at 95 °C for 1 h. The absorbance was measured using a spectrophotometer at a wavelength of 532 nm. Trolox (100 μM), an antioxidant analogous to vitamin E, served as a positive control. The results were reported as nmol TBARS per gram of tissue. A parallel TBARS experiment was also conducted using a standard alkyl aryl selenide (2-((phenylselanyl)­methyl)­pyridine), dissolved in DMSO at the same concentrations tested, ranging from 25 to 500 μM.

#### 3.3.2 Protein Carbonylation

The evaluation of protein carbonylation was performed according to a previously established protocol,^74^ using freshly prepared brain homogenate diluted at a 1:8 ratio in Tris-HCl buffer. Test compounds (10 μL), ranging in concentrations from 25 to 200 μM, were individually introduced into separate test tubes. Trolox (100 μM) served as the standard antioxidant. The diluted brain homogenate (940 μL) was then added to all tubes, followed by SNP (1 mM) as the inducing oxidative agent (50 μL). The tubes were promptly incubated at 37 °C for 2 h. Following incubation, 2 M HCl was added to blank tubes (200 μL), while 10 mM 2,4-dinitrophenyl-hydrazine (DNPH) solution was added to test tubes (200 μL). The mixture was incubated for 1 h in darkness at room temperature, with intermittent stirring every 15 min to facilitate the color reaction. Subsequently, 500 μL of denaturation buffer, 1.5 mL of ethanol, and 1.5 mL of hexane were added to all tubes, which were then vigorously shaken for 40 s. The tubes were then centrifuged at 900*g* for 15 min, and the supernatant was carefully decanted. The resulting pellet was washed twice with 1 mL of ethanol/ethyl acetate solution (1:1). After allowing the pellet to dry air for 2 min, it was resuspended in 1 mL of denaturation buffer and subjected to spectrophotometric analysis at 370 nm. The findings were quantified as nmol carbonyl per gram of tissue.

#### 3.3.3 Cerebral Monoamine Oxidase (MAO) Activity

Mitochondria-enriched brain preparations were obtained in accordance with established methodology outlined in literature.^75^ The homogenate was prepared using the mouse brain tissue and a homogenization buffer (Na_2_PO_4_/KH_2_PO_4_/sucrose) at a ratio of 1:4 (w/v). The homogenate was centrifuged at 900*g* at 4 °C for 5 min. The resulting supernatant was separated and subjected to centrifugation at 12,500*g* for 15 min. The resulting pellet was resuspended in the homogenization buffer and subjected to a further round of centrifugation under identical conditions. Finally, the mitochondrial pellet obtained was reconstituted in an assay buffer solution (Na_2_PO_4_/KH_2_PO_4_/KCl). Protein concentration was estimated using the Bradford method^76^ and standardized as 1 mg/mL for the experiments.

MAO activity was assessed following the method outlined by Krajl,^77^ with some modifications by Matsumoto.^78^ Briefly, a 100 μL aliquot of the mitochondria-rich fraction was incubated at 37 °C for 5 min with specific MAO inhibitors: clorgyline (250 nM, a MAO-A inhibitor), or pargyline (250 nM, a MAO-B inhibitor). Then, 10 μL of each compound was introduced at varying concentrations (25–200 μM). Water (10 μL) was used in the control tubes. Afterward, the substrate, 20 μL of kynuramine dihydrobromide, was added to achieve a final concentration of 90 μM for MAO-A and 60 μM for MAO-B. The samples were incubated at 37 °C for 30 min. The reaction was halted by the addition of 300 μL of 10% trichloroacetic acid (TCA). After centrifuging at 16,000*g* for 15 min, an aliquot of 800 μL of the supernatant was mixed with 1 mL of 1 M sodium hydroxide (NaOH). The samples were measured using a fluorimeter with excitation at 315 nm and emission at 380 nm.

### 3.4 In Vivo Experiments

Female mice were used to evaluate acute oral toxicity (*n* = 6 mice per group), while male mice were used for behavioral assays. For behavioral experiments, animals were randomly divided into different experimental groups (*n* = 7–11 mice per group).

#### 3.4.1 Screening of Antidepressant-like Effects in the Mouse TST

A behavioral screening was conducted using the three salts to determine their potential antidepressant-like actions (*n* = 7–9 mice/group). The animals were divided into five experimental groups (*n* = 8–10/group): (I) Control, receiving the vehicle (water), (II) Compound **3A**, (III) Compound **3B**, (IV) Compound **3C**, and (V) Fluoxetine (a positive control).

A dosage of 5 mg/kg (intragastric route, i.g., by gavage) was administered to all groups receiving the salts. Fluoxetine, a standard antidepressant of selective serotonin reuptake inhibitor (SSRI) class, was administered at a dose of 20 mg/kg, by intraperitoneal (i.p.) route.^79^ The TST was conducted 30 min following drug administration, with the OFT being performed 4 min prior to the TST.

#### 3.4.2 Time-Response Curve Experiment

Following the selection of the most promising salt during screening (Compound **3B**), an analysis of the compound’s time-response curve was conducted in the mouse TST. For this purpose, the animals were divided into five groups: (I) control (water; i.g.), (II) the group receiving compound **3B** 15 min prior to TST, (III) the group receiving compound **3B** 30 min before TST, (IV) the group receiving compound **3B** 60 min before TST, and (V) the group receiving compound **3B** 120 min before TST. The dosage of salt was standardized at 5 mg/kg (i.g). OFT was conducted 4 min prior to the TST.

#### 3.4.3 Dose–Response Curve Experiment

After choosing a suitable preadministration time, the dose–response curve of the compound at this fixed time was analyzed. The animals were categorized into six groups: I) a control group (administered water; i.g.) and groups II, III, IV and V) receiving compound **3B** at doses of 0.5 mg/kg, 1 mg/kg, 2.5 mg/kg, and 5 mg/kg (i.g.), respectively. Additionally, there was a fluoxetine group (VI), serving as a positive control, at a dosage of 20 mg/kg; i.p. route. Thirty minutes postadministration, the animals underwent the TST. Four minutes prior to the TST, they underwent the OFT.

### 3.5 Oral Acute Toxicity Protocol

The evaluation of the acute toxicity of compounds **3A** and **3B** via oral administration was conducted in female Swiss mice, following the guidelines outlines in Protocol 423–OECD. The acute oral toxicity study of compound 3**C** was previously conducted by our research group, showing low toxicity^22^ and therefore was not evaluated here.

For the toxicological experiment, conducted in distinct steps as suggested by the OECD protocol, a total of 36 animals were used. The animals were divided into 3 experimental groups (*n* = 6 mice/group) for each dosage testing: (I) Control, (II) Compound **3A**, and (III) Compound **3B**. Both compounds (**3A** and **3B**) were administered at a single dose of 50 or 300 mg/kg via gavage, while control animals received water (vehicle). This high dose (300 mg/kg) was fractionated into three oral administrations of 100 mg/kg at a volume of 10 mL/kg with an interval of 30 min each. Over a period of 14 days, water and food consumption were monitored. In addition, changes in body weight and behavioral parameters such as lethargy, piloerection, and diarrhea were observed. Following this evaluation period, locomotion and exploration of the animals were assessed using the OFT. Subsequently, the animals were euthanized by an overdose of isoflurane, and blood, brain, liver, and kidney samples were collected for ex vivo analysis.

Blood plasma was used to assess liver and kidney function markers by quantifying the activity of AST and ALT, as well as urea levels, using commercially available kits (LABTEST; BIOCLIN). The brain, liver and kidneys were employed to investigate potential oxidative damage to lipids as a toxicity parameter utilizing the TBARS technique.^73^

### 3.6 Behavioral Tests

The assessment of behavioral tests occurred within an acoustic and visually isolated environment. These tests, containing all experimental groups, were conducted and reproduced in different days (2 sets). Prior to commencing any experimental set, the animals underwent 1 h of acclimatization. To prevent cross-contamination, the behavioral apparatuses were thoroughly cleaned with 20% alcohol after each mouse. All tests were recorded using a Microsoft Lifecam HD-3000, and each video recording was subsequently reviewed by a trained observer who was blinded to the experimental conditions.

#### 3.6.1 OFT

The aim of this behavioral test is to assess the locomotion and exploration behavior of the animals using an open field apparatus measuring 30 cm × 30 cm × 15 cm, constructed from plywood with a floor divided into nine quadrants, facilitating free movement for the animals. During the OFT, the animals were positioned in the center of the apparatus and subjected to a 5 min testing period, following the protocol outlined in literature.^80^ The number of quadrant crossings and rearings was recorded and quantified during testing phase.

#### 3.6.2 TST

This test aims to assess depressive-like behavior and antidepressant-like action of therapies by measuring immobility. An increase in immobility signifies a behavioral response indicative of despair, while a decrease in immobility suggests antidepressant-like effects.^81^ The test was conducted using a mouse suspension box made of wood, measuring 50 cm × 21 cm × 21 cm, designed to prevent any visual or physical interaction among the animals. A suspension bar was positioned atop the box, and the mouse’s tail was affixed to the bar using adhesive tape. The test duration was 6 min, during which the animal’s latency to the first immobility episode and the total duration of immobility (sec) were assessed.

### 3.7 Statistical Analysis

GraphPad Prism software version 8.02 was utilized for statistical analysis. Comparisons among experimental groups were conducted using one-way analysis of variance (ANOVA). In vivo data, data normality was tested. Parametric tests were applied to normal data and nonparametric tests were applied to nonnormal data. The Dunnett post hoc test was used for parametric data and Kruskal–Wallis for nonparametric data. For in vitro data, Tukey’s post hoc test was performed. Results are presented as the mean ± standard error of the mean (S.E.M.). Statistical significance was considered at *p* < 0.05.

## Supplementary Material



## Notes

In vitro TBARS results; OFT data; ex vivo TBARS (toxicity) results; in silico ADMET profiles; and compounds’ characterization data (salts **3A**, **3B,** and **3C**) (PDF)

## Abbreviations

ADMET: absorption, distribution, metabolism, excretion, and toxicity
ALT: alanine aminotransferase
ANOVA: analysis of variance
AST: aspartate aminotransferase
C: control group
CONCEA: National Council for the Control of Animal Experimentation
DNPH: 2,4-dinitrophenyl-hydrazine
FLX: fluoxetine group
FST: forced swim test
i.g.: intragastric
i.p.: intraperitoneal
IC_50_: 50% inhibitory concentration
LASOL: Clean Organic Synthesis Laboratory
LD_50_: median lethal dose
MAO: monoamine oxidase
NaOH: sodium hydroxide
OFT: open field test
SEM.: mean ± standard error of the mean
SDS: sodium dodecyl sulfate
SNP: sodium nitroprusside
SSRI: selective serotonin reuptake inhibitor
TBA: thiobarbituric acid
TBARS: thiobarbituric acid reactive substances
TCA: trichloroacetic acid
TST: tail suspension test
WHO: World Health Organization
