# Supplementary material for: Antidepressant-like Properties of a Selenium-Containing Pyridinium Salt Explored through In Vitro, In Vivo, and In Silico Approaches
Source: ACS Chem Neurosci. 2025 Sep 12;16(19):3713–27. doi: 10.1021/acschemneuro.5c00233 (PMC12498411; doi:10.1021/acschemneuro.5c00233)
Supplement: Supplementary file 1 [file cn5c00233_si_001.pdf]

SUPPLEMENTARY MATERIAL

Antidepressant-like properties of a selenium-containing pyridinium salt explored through in vitro, in vivo, and in silico approaches

Mariana Parron Paim[a], Taís da Silva Teixeira Rech[a], Letícia Devantier Krüger[a], Larissa Sander Magalhães[a], Filipe Penteado[b], Caroline Signorini Gomes[b], Eder João Lenardão[b], César Augusto Brüning[a]*, Cristiani Folharini Bortolatto*

This supplementary material includes:

- In vitro TBARS results (Figure S1);
- OFT data (Figures S2–S4);
- Ex vivo TBARS (toxicity) results (Figure S5);
- In silico ADMET profiles (Tables S1–S3);
- Compounds' characterization data (salts **3A**, **3B** and **3C**).

SUPPLEMENTARY FIGURES

- **In vitro TBARS results (Figure S1)**

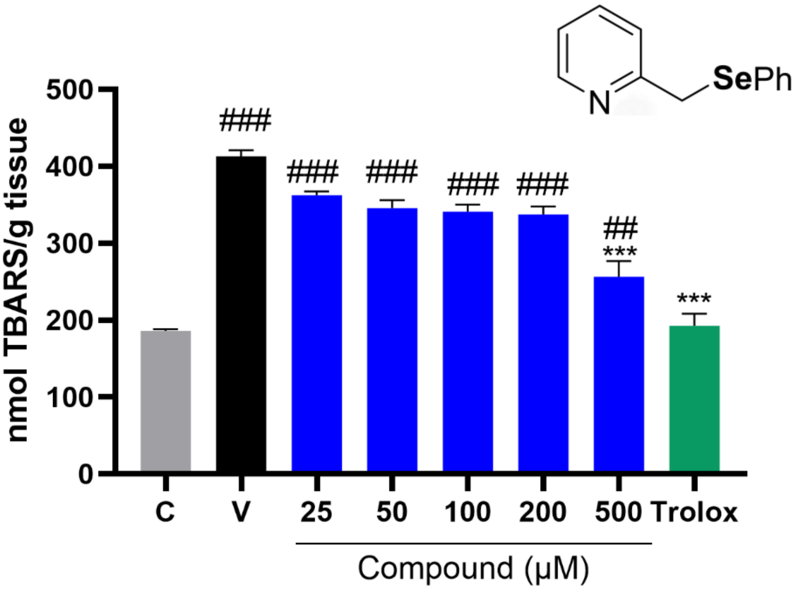

Figure S1. Effects of 2-((phenylselanyl)methyl)pyridine compound (5-500 μM, an alkyl aryl selenide used to compare with the salts) on brain lipid peroxidation (TBARS) level in vitro. Three independent experiments (n = 3) were performed in duplicate. Trolox was used as positive control and tested at a concentration of 100 μM. A one-way ANOVA followed by Tukey's post hoc test was applied, and the results were expressed as nmol of TBARS per gram of tissue. ##p<0.01 and ###p<0.001 compared with the control. ***p<0.001 compared with Vehicle (V, DMSO+SNP). Sodium nitroprusside (SNP) was used as an inducer of lipid peroxidation.

- **OFT data (Figures S2–S4)**

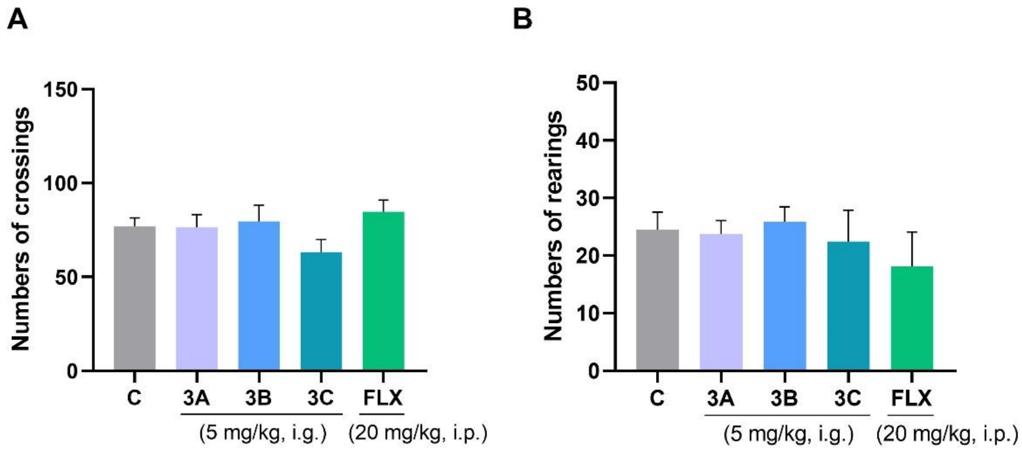

Figure S2. Locomotor (A) and exploratory (B) activities of male mice treated with compounds **3A**, **3B** and **3C** (5 mg/kg, i.g.) in the OFT. The results represent the mean ± S.E.M. of 8-11 mice/group. Data analyses were carried out by the one-way ANOVA test. Abbreviations: C = control and FLX = fluoxetine.

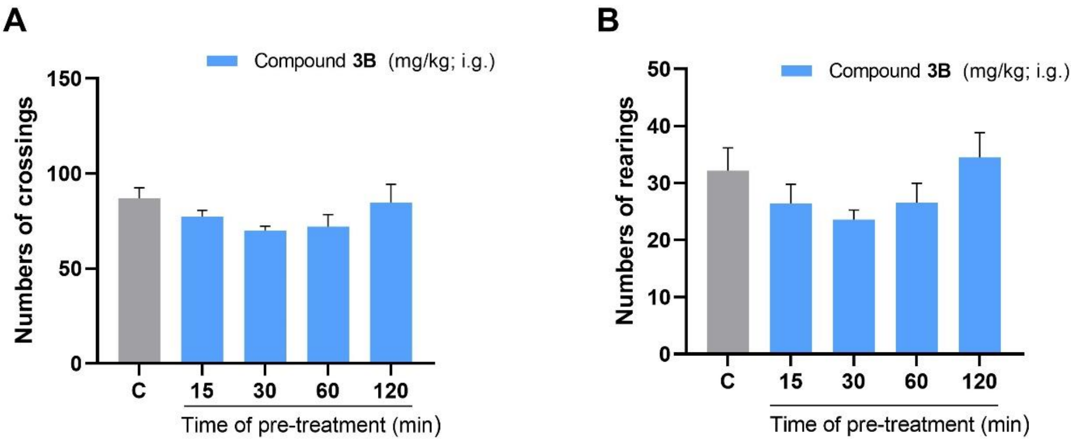

Figure S3. Locomotor (A) and exploratory (B) activities of male mice treated with compound **3B** in a time-response curve (15-120 min) in the OFT. The results represent the mean ± S.E.M. of 8-11 mice/group. Data analyses were carried out by the one-way ANOVA test. Abbreviations: C = control.

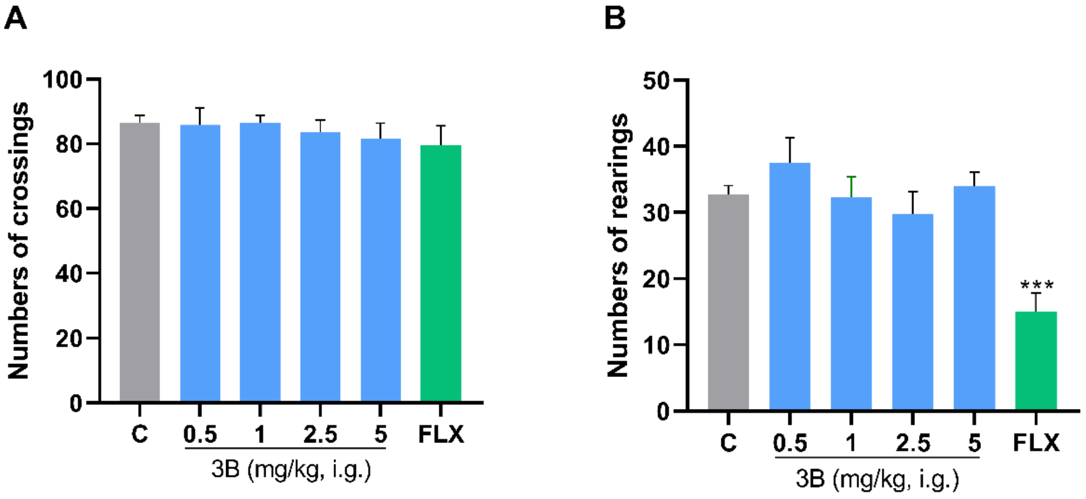

Figure S4. Locomotor (A) and exploratory (B) activities of male mice treated with compound **3B** in a dose-response curve in the OFT. The results represent the mean ± S.E.M. of 8-11 mice/group. Data analyses were carried out by the one-way ANOVA/Dunnett's test. ***p<0.001 in relation to the control group. Abbreviations: C = control and FLX = fluoxetine.

- **Ex vivo TBARS (toxicity) results**

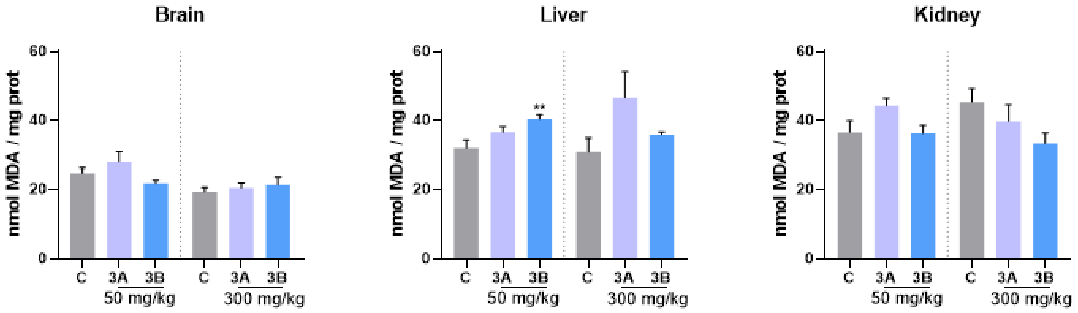

Figure S5. Lipoperoxidation levels in biological tissues of female mice exposed to an oral acute administration of compound **A** and **B** (50 and 300 mg/kg, i.g.). Analyses were performed in the brain (A), liver (B), and kidneys (C) according to the toxicity protocol. Data analyses were carried out by the one-way ANOVA/Dunnett's test. **$p<0.01$ in relation to the control group. Values are expressed as mean ± SEM. Abbreviations: C = control group.

SUPPLEMENTARY TABLES

- **In silico ADMET profiles**

Table S1. The table shows the data obtained by the in silico pharmacokinetic properties of compound **3A**.

Descriptor (compound 3A)	Value
Molecular Weight	447.234
LogP	-0.6093
#Rotatable Bonds	6
#Acceptors	1
#Donors	0
Surface Area	159.025

Property	Model Name	Predicted Value	Unit
Absorption	Water solubility	-2.588	Numeric (log mol/L)
Absorption	Caco2 permeability	1.062	Numeric (log Papp in 10 cm/s)
Absorption	Intestinal absorption (human)	73.435	Numeric (% Absorbed)
Absorption	Skin Permeability	-2.593	Numeric (log Kp)
Absorption	P-glycoprotein substrate	Yes	Categorical (Yes/No)
Absorption	P-glycoprotein I inhibitor	Yes	Categorical (Yes/No)
Absorption	P-glycoprotein II inhibitor	Yes	Categorical (Yes/No)
Distribution	VDss (human)	0.495	Numeric (log L/kg)
Distribution	Fraction unbound (human)	0.186	Numeric (Fu)
Distribution	BBB permeability	0.496	Numeric (log BB)
Distribution	CNS permeability	-1.209	Numeric (log PS)

Metabolism	CYP2D6 substrate	No	Categorical (Yes/No)
Metabolism	CYP3A4 substrate	Yes	Categorical (Yes/No)
Metabolism	CYP1A2 inhibitor	Yes	Categorical (Yes/No)
Metabolism	CYP2C19 inhibitor	Yes	Categorical (Yes/No)
Metabolism	CYP2C9 inhibitor	No	Categorical (Yes/No)
Metabolism	CYP2D6 inhibitor	Yes	Categorical (Yes/No)
Metabolism	CYP3A4 inhibitor	No	Categorical (Yes/No)
Excretion	Total Clearance	2.464	Numeric (log ml/min/kg)
Excretion	Renal OCT2 substrate	Yes	Categorical (Yes/No)
Toxicity	AMES toxicity	Yes	Categorical (Yes/No)
Toxicity	Max. tolerated dose (human)	0.283	Numeric (log mg/kg/day)
Toxicity	hERG I inhibitor	No	Categorical (Yes/No)
Toxicity	hERG II inhibitor	Yes	Categorical (Yes/No)
Toxicity	Oral Rat Acute Toxicity (LD50)	1.66	Numeric (mol/kg)
Toxicity	Oral Rat Chronic Toxicity (LOAEL)	-0.874	Numeric (log mg/kg_bw/day)
Toxicity	Hepatotoxicity	No	Categorical (Yes/No)
Toxicity	Skin Sensitisation	No	Categorical (Yes/No)
Toxicity	T.Pyriformis toxicity	0.824	Numeric (log ug/L)
Toxicity	Minnow toxicity	1.078	Numeric (log mM)

Table S2. The table shows the data obtained by in silico pharmacokinetic properties of compound **3B**.

**Descriptor (compound 3B)**	**Value**
Molecular Weight	461.261
LogP	-0.30088
#Rotatable Bonds	6
#Acceptors	1
#Donors	0
Surface Area	165.390

**Property**	**Model Name**	**Predicted Value**	**Unit**
Absorption	Water solubility	-2.451	Numeric (log mol/L)
Absorption	Caco2 permeability	0.924	Numeric (log Papp in 10 cm/s)
Absorption	Intestinal absorption (human)	73.538	Numeric (% Absorbed)
Absorption	Skin Permeability	-2.508	Numeric (log Kp)
Absorption	P-glycoprotein substrate	Yes	Categorical (Yes/No)
Absorption	P-glycoprotein I inhibitor	Yes	Categorical (Yes/No)
Absorption	P-glycoprotein II inhibitor	Yes	Categorical (Yes/No)
Distribution	VDss (human)	0.831	Numeric (log L/kg)
Distribution	Fraction unbound (human)	0.143	Numeric (Fu)
Distribution	BBB permeability	0.516	Numeric (log BB)
Distribution	CNS permeability	-1.262	Numeric (log PS)
Metabolism	CYP2D6 substrate	No	Categorical (Yes/No)
Metabolism	CYP3A4 substrate	Yes	Categorical (Yes/No)

Metabolism	CYP1A2 inhibitor	Yes	Categorical (Yes/No)
Metabolism	CYP2C19 inhibitor	No	Categorical (Yes/No)
Metabolism	CYP2C9 inhibitor	No	Categorical (Yes/No)
Metabolism	CYP2D6 inhibitor	Yes	Categorical (Yes/No)
Metabolism	CYP3A4 inhibitor	Yes	Categorical (Yes/No)
Excretion	Total Clearance	2.518	Numeric (log ml/min/kg)
Excretion	Renal OCT2 substrate	Yes	Categorical (Yes/No)
Toxicity	AMES toxicity	No	Categorical (Yes/No)
Toxicity	Max. tolerated dose (human)	0.4	Numeric (log mg/kg/day)
Toxicity	hERG I inhibitor	No	Categorical (Yes/No)
Toxicity	hERG II inhibitor	Yes	Categorical (Yes/No)
Toxicity	Oral Rat Acute Toxicity (LD50)	1.905	Numeric (mol/kg)
Toxicity	Oral Rat Chronic Toxicity (LOAEL)	-0.783	Numeric (log mg/kg_bw/day)
Toxicity	Hepatotoxicity	No	Categorical (Yes/No)
Toxicity	Skin Sensitisation	No	Categorical (Yes/No)
Toxicity	T.Pyriformis toxicity	0.359	Numeric (log ug/L)
Toxicity	Minnow toxicity	0.475	Numeric (log mM)

Table S3. The table shows the data obtained by in silico pharmacokinetic properties of compound **3C**.

Descriptor (compound 3C)	Value
Molecular Weight	481.679
LogP	0.0441
#Rotatable Bonds	6
#Acceptors	1
#Donors	0
Surface Area	169.328

Property	Model Name	Predicted Value	Unit
Absorption	Water solubility	-3.022	Numeric (log mol/L)
Absorption	Caco2 permeability	1.052	Numeric (log Papp in 10^{-6} cm/s)
Absorption	Intestinal absorption (human)	77.434	Numeric (% Absorbed)
Absorption	Skin Permeability	-2.605	Numeric (log Kp)
Absorption	P-glycoprotein substrate	Yes	Categorical (Yes/No)
Absorption	P-glycoprotein I inhibitor	Yes	Categorical (Yes/No)
Absorption	P-glycoprotein II inhibitor	Yes	Categorical (Yes/No)
Distribution	VDss (human)	0.56	Numeric (log L/kg)
Distribution	Fraction unbound (human)	0.196	Numeric (Fu)
Distribution	BBB permeability	0.495	Numeric (log BB)
Distribution	CNS permeability	-1.209	Numeric (log PS)
Metabolism	CYP2D6 substrate	No	Categorical (Yes/No)

Metabolism	CYP3A4 substrate	Yes	Categorical (Yes/No)
Metabolism	CYP1A2 inhibitor	Yes	Categorical (Yes/No)
Metabolism	CYP2C19 inhibitor	Yes	Categorical (Yes/No)
Metabolism	CYP2C9 inhibitor	No	Categorical (Yes/No)
Metabolism	CYP2D6 inhibitor	Yes	Categorical (Yes/No)
Metabolism	CYP3A4 inhibitor	No	Categorical (Yes/No)
Excretion	Total Clearance	2.51	Numeric (log ml/min/kg)
Excretion	Renal OCT2 substrate	Yes	Categorical (Yes/No)
Toxicity	AMES toxicity	No	Categorical (Yes/No)
Toxicity	Max. tolerated dose (human)	0.176	Numeric (log mg/kg/day)
Toxicity	hERG I inhibitor	No	Categorical (Yes/No)
Toxicity	hERG II inhibitor	Yes	Categorical (Yes/No)
Toxicity	Oral Rat Acute Toxicity (LD50)	1.741	Numeric (mol/kg)
Toxicity	Oral Rat Chronic Toxicity (LOAEL)	1.647	Numeric (log mg/kg_bw/day)
Toxicity	Hepatotoxicity	No	Categorical (Yes/No)
Toxicity	Skin Sensitisation	No	Categorical (Yes/No)
Toxicity	T.Pyriformis toxicity	0.865	Numeric (log µg/L)
Toxicity	Minnow toxicity	0.579	Numeric (log mM)

COMPOUNDS' CHARACTERIZATION DATA

- **Salt 3A characterization**

Name: 1-(2-oxo-2-phenylethyl)-2-((phenylselanyl)methyl)pyridin-1-ium bromide.

^{1}H NMR (DMSO-d^{6}, 400 MHz) δ (ppm) 8.97 – 8.95 (m, 1H); 8.38 – 8.35 (m, 1H); 8.15 (d, J = 7.4 Hz, 2H); 8.05 – 8.02 (m, 1H); 7.83 – 7.79 (m, 1H); 7.70 – 7.67 (m, 2H); 7.52 (d, J = 7.7 Hz, 1H); 7.40 – 7.26 (m, 5H); 6.61 (s, 2H); 4.72 (s, 2H). ^{13}C NMR (DMSO-d^{6}, 100 MHz) δ (ppm) 190.3, 155.2, 148.2, 154.5, 135.0, 134.6, 133.7, 129.3, 129.1, 129.0, 128.8, 128.6, 126.0, 125.7, 62.9, 26.7.

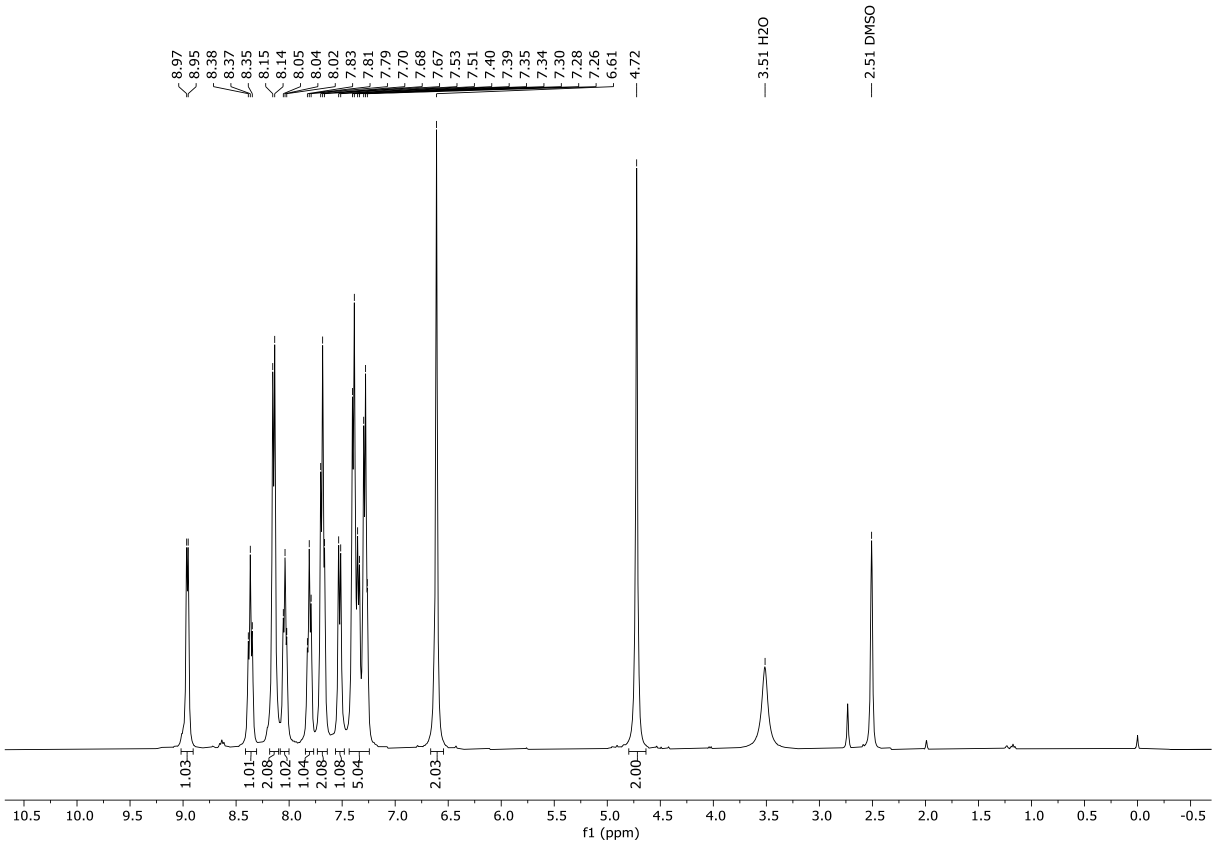

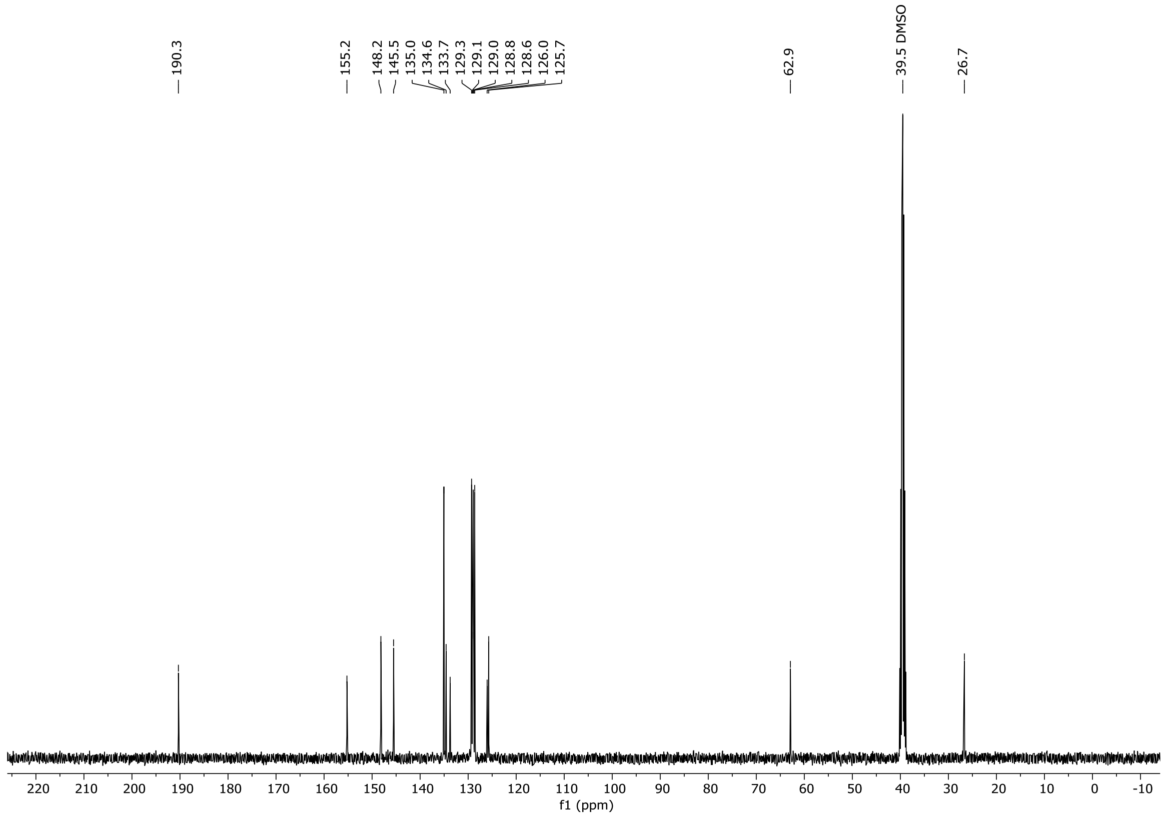
190.3
155.2
148.2
145.5
135.0
134.6
133.7
129.3
129.1
129.0
128.8
128.6
126.0
125.7
62.9
39.5 DMSO
26.7
220 210 200 190 180 170 160 150 140 130 120 110 100 90 80 70 60 50 40 30 20 10 0 -10
f1 (ppm)

- **Salt 3B characterization**

Name: 1-(2-oxo-2- (p-tolyl)ethyl)-2-((phenylselanyl)methyl)pyridin-1-ium bromide.

^{1}H NMR (DMSO-d^{6}, 400 MHz) *δ* (ppm) 9.04 – 9.02 (m, 1H); 8.50 (td, *J* = 7.8, 1.2 Hz, 1H); 8.14 – 8.07 (m, 3 H); 7.83 (dd, *J* = 8.0, 1.0 Hz, 1H); 7.50 (d, *J* = 8.0 Hz, 2H); 7.33-7.27 (m, 5H); 6.68 (s, 2H); 4.88 (s, 2H); 2.46 (s, 3H). ^{13}C NMR (DMSO-d^{6}, 100 MHz) *δ* (ppm) 190.0, 153.6, 148.7, 146.0, 145.4, 131.8, 131.2, 131.1, 129.6, 129.4, 129.2, 128.7, 128.2 126.2, 62.9, 34.8, 21.3.

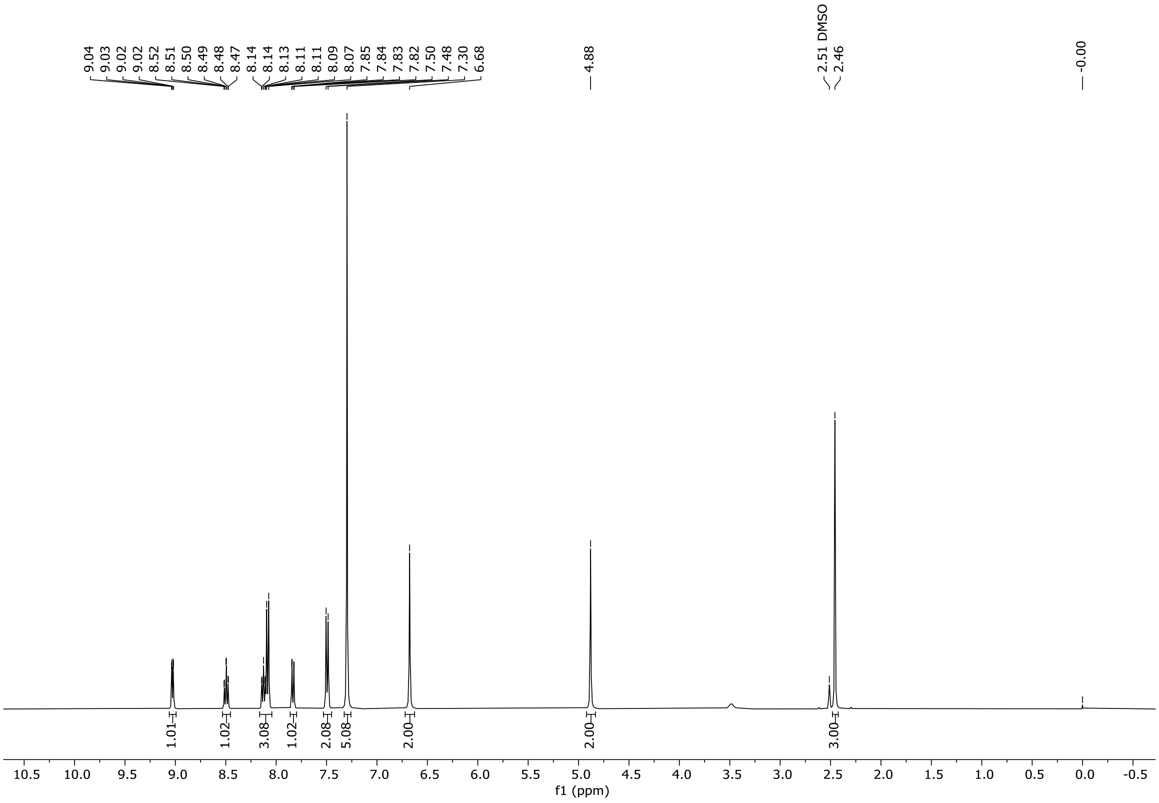

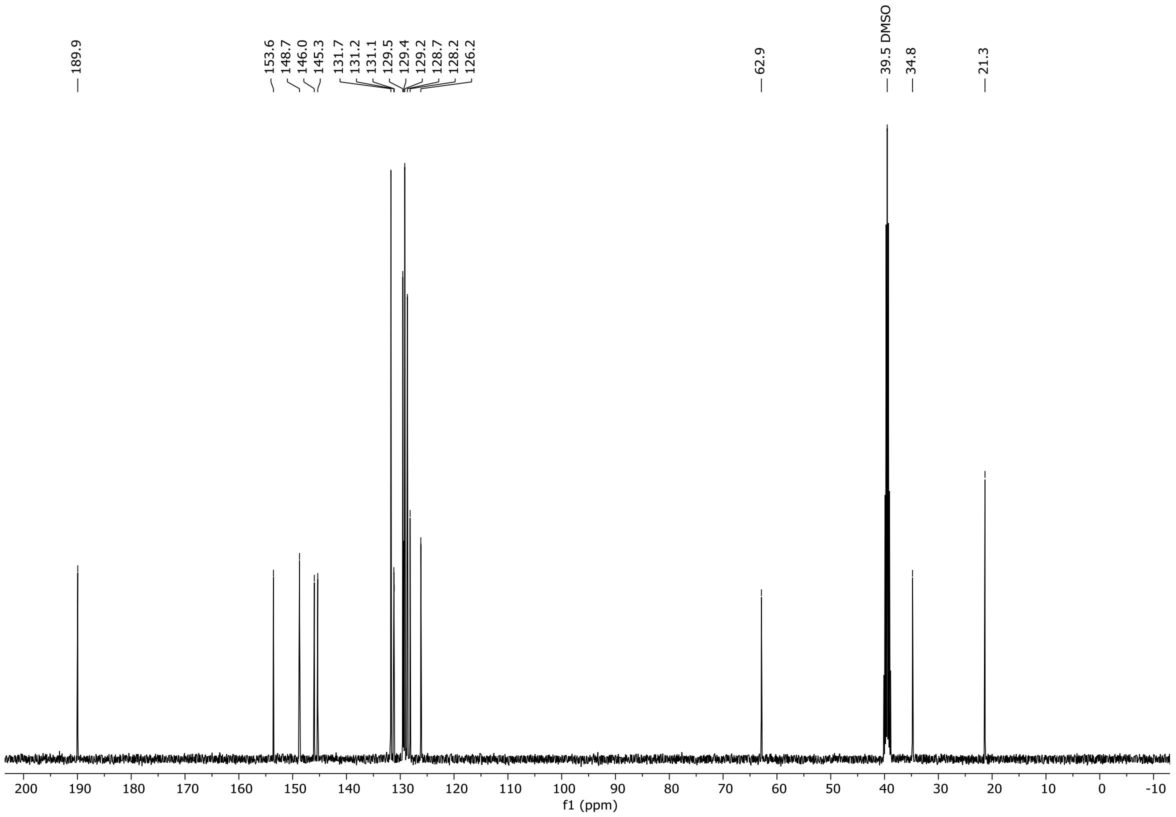
189.9
153.6
148.7
146.0
145.3
131.7
131.2
131.1
129.5
129.4
129.2
128.7
128.2
126.2
62.9
39.5 DMSO
34.8
21.3
200 190 180 170 160 150 140 130 120 110 100 90 80 70 60 50 40 30 20 10 0 -10
f1 (ppm)

- **Salt 3C characterization**

Name: 1-(2-(4-chlorophenyl)-2-oxoethyl)-2-((phenylselanyl)methyl)pyridin-1-ium bromide.

^{1}H NMR (DMSO-d^{6}, 400 MHz) δ (ppm) 8.97 (d, J = 5.9 Hz, 1H); 8.37 (t, J = 7.5 Hz, 1H); 8.17 (d, J = 8.5 Hz, 2H); 8.06 – 8.03 (m, 1H); 7.77 (d, J = 8.5 Hz, 2H); 7.53 (d, J = 7.7 Hz, 1H); 7.40 – 7.26 (m, 5H); 6.63 (s, 2H); 4.74 (s, 2H). ^{13}C NMR (DMSO-d^{6}, 100 MHz) δ (ppm) 189.5, 155.2, 148.2, 145.6, 139.4, 135.0, 132.5, 130.5, 129.3, 129.1, 129.1, 128.8, 126.0, 125.7. 62.9, 26.7.

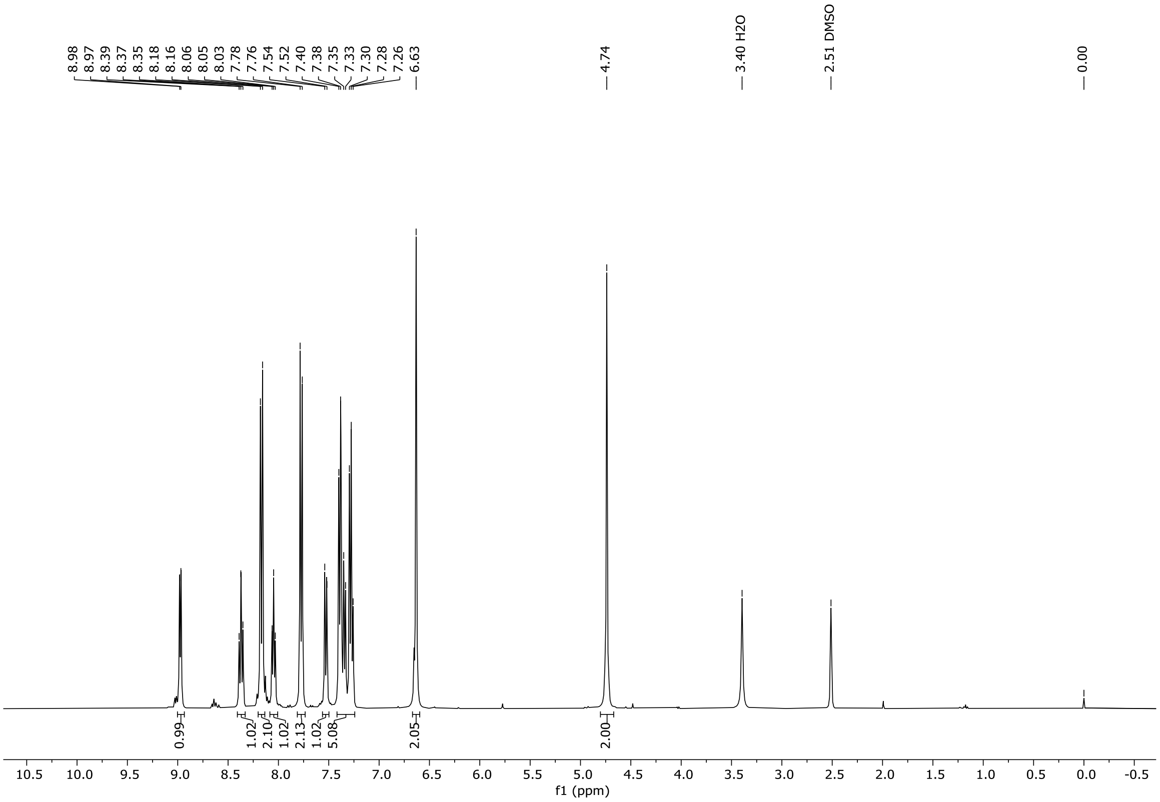

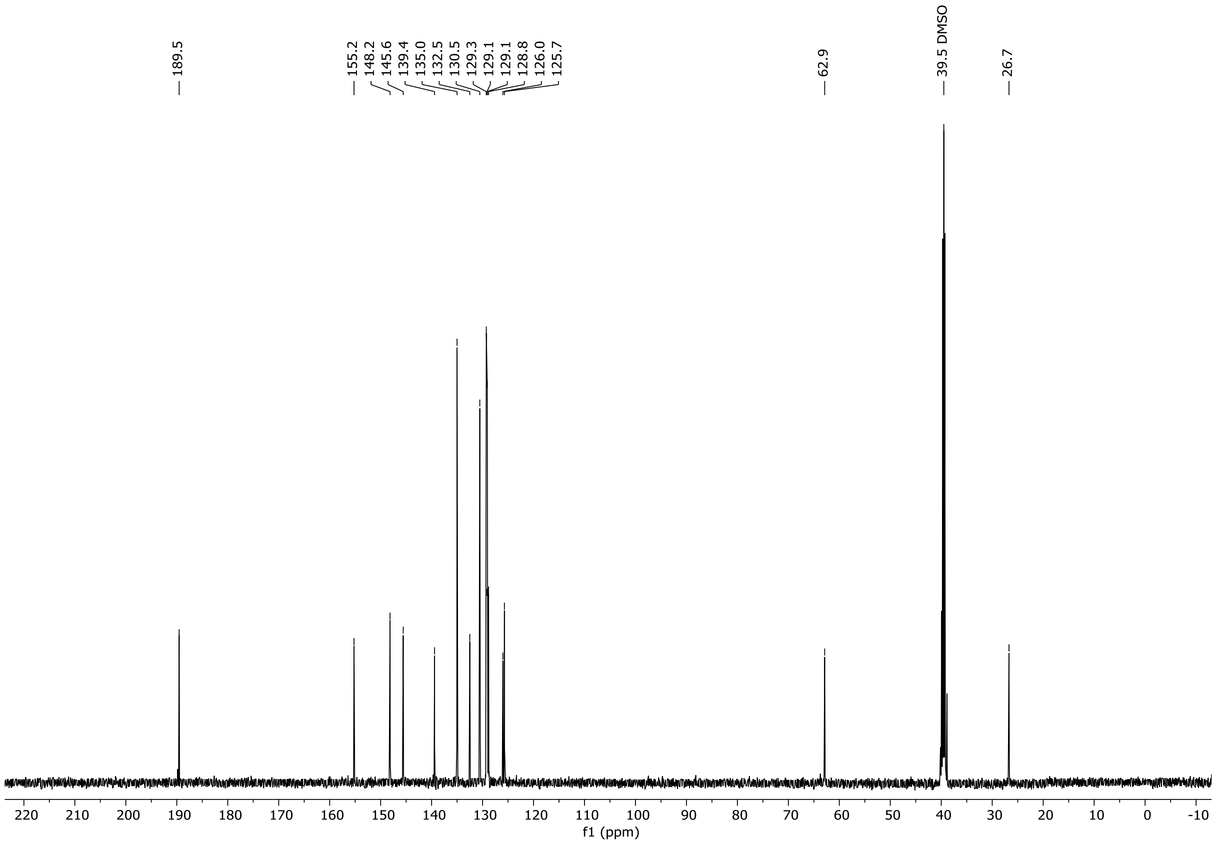

189.5
155.2
148.2
145.6
139.4
135.0
132.5
130.5
129.3
129.1
129.1
128.8
126.0
125.7
62.9
39.5 DMSO
26.7
220 210 200 190 180 170 160 150 140 130 120 110 100 90 80 70 60 50 40 30 20 10 0 -10
f1 (ppm)
